# IgG Fc-binding motif-conjugated HIV-1 fusion inhibitor exhibits improved potency and *in vivo* half-life: Potential application in combination with broad neutralizing antibodies

**DOI:** 10.1371/journal.ppat.1008082

**Published:** 2019-12-05

**Authors:** Wenwen Bi, Wei Xu, Liang Cheng, Jing Xue, Qian Wang, Fei Yu, Shuai Xia, Qi Wang, Guangming Li, Chuan Qin, Lu Lu, Lishan Su, Shibo Jiang

**Affiliations:** 1 Key Laboratory of Medical Molecular Virology of MOE/NHC/CAMS, School of Basic Medical Sciences and Shanghai Public Health Clinical Center, Fudan University, Shanghai, China; 2 Lineberger Comprehensive Cancer Center, University of North Carolina at Chapel Hill, Chapel Hill, North Carolina, United States of America; 3 Key Laboratory of Human Disease Comparative Medicine, Chinese Ministry of Health, Beijing Key Laboratory for Animal Models of Emerging and Re-emerging Infectious Diseases, Institute of Laboratory Animal Science, Chinese Academy of Medical Sciences and Comparative Medicine Center, Peking Union Medical College, Beijing, China; 4 Lindsley F. Kimball Research Institute, New York Blood Center, New York, New York, United States of America; King's College London, UNITED KINGDOM

## Abstract

The clinical application of conventional peptide drugs, such as the HIV-1 fusion inhibitor enfuvirtide, is limited by their short half-life *in vivo*. To overcome this limitation, we developed a new strategy to extend the *in vivo* half-life of a short HIV-1 fusion inhibitory peptide, CP24, by fusing it with the human IgG Fc-binding peptide (IBP). The newly engineered peptide IBP-CP24 exhibited potent and broad anti-HIV-1 activity with IC_50_ values ranging from 0.2 to 173.7 nM for inhibiting a broad spectrum of HIV-1 strains with different subtypes and tropisms, including those resistant to enfuvirtide. Most importantly, its half-life in the plasma of rhesus monkeys was 46.1 h, about 26- and 14-fold longer than that of CP24 (t_1/2_ = 1.7 h) and enfuvirtide (t_1/2_ = 3 h), respectively. IBP-CP24 intravenously administered in rhesus monkeys could not induce significant IBP-CP24-specific antibody response and it showed no obvious *in vitro* or *in vivo* toxicity. In the prophylactic study, humanized mice pretreated with IBP-CP24 were protected from HIV-1 infection. As a therapeutic treatment, coadministration of IBP-CP24 and normal human IgG to humanized mice with chronic HIV-1 infection resulted in a significant decrease of plasma viremia. Combining IBP-CP24 with a broad neutralizing antibody (bNAb) targeting CD4-binding site (CD4bs) in gp120 or a membrane proximal external region (MPER) in gp41 exhibited synergistic effect, resulting in significant dose-reduction of the bNAb and IBP-CP24. These results suggest that IBP-CP24 has the potential to be further developed as a new HIV-1 fusion inhibitor-based, long-acting anti-HIV drug that can be used alone or in combination with a bNAb for treatment and prevention of HIV-1 infection.

## Author summary

Enfuvirtide (T20) is the first US FDA-approved anti-HIV peptide drug. However, its clinical application is limited because of its short half-life and emergence of T20-resistant HIV strains. Here we developed a new strategy to prolong the half-life of a short anti-HIV peptide (CP24) by conjugating it with the human IgG Fc-binding peptide (IBP). IBP-CP24 exhibited potent and broad anti-HIV-1 activity and prolonged half-life, indicating its potential to be developed as a long-acting anti-HIV drug. Interestingly, combinational use of IBP-CP24 with a broad HIV neutralizing antibody, such as N6, showed synergistic anti-HIV-1 effect, suggesting that IBP-CP24 can be used together with N6 to treat HIV-1 infection because N6, as a biomissile carrying IBP-CP24, binds gp120 to make the first strike, and releases IBP-CP24 that binds gp41 to make the second strike to HIV-1. Therefore, combining IBP-CP24 with a bNAb may reduce the dose of the antibody and peptide, thus the cost of the treatment.

## Introduction

Acquired immune deficiency syndrome (AIDS) caused by human immunodeficiency virus (HIV) infection continues to be a major global public health issue. In 2017, the Joint United Nations Programme in HIV and AIDS (UNAIDS) reported that about 36.9 million people were living with HIV globally, around 1.8 million people became newly infected with HIV and approximately 0.9 million people died from AIDS-related illnesses (http://www.unaids.org). Currently, no effective vaccine is available to prevent HIV-1 infection. Despite the success of combination anti-retroviral therapy (cART), challenges remain in the management of chronic HIV-1 infection. Therapies that combine reverse-transcriptase inhibitors (RTIs) and protease inhibitors have shown such problems as adherence, emergence of drug-resistance, and toxic side effects with long-term treatment [1–2]. Moreover, such therapies could not prevent HIV-1 from entry into target cells. However, HIV-1 infection of target cells can be efficiently suppressed by fusion inhibitors derived from HIV-1 gp41 to target the virus entry step. Thus, fusion inhibitors are becoming an attractive approach for intervention in the early viral life cycle. Currently, enfuvirtide (Fuzeon or T20) is the first clinically approved HIV-1 fusion inhibitor [3–6]. However, the clinical application of T20 is limited by its short plasma half-life and tendency to develop drug-resistance [7–10], highlighting the importance of developing long-acting anti-HIV fusion inhibitors.

Although a few strategies have been developed to improve the pharmacokinetics (PK) of protein drugs, challenges remain in the modification of small peptides to improve their half-life, while still maintaining their safety and activity. PEGylation is commonly used to increase *in viv*o protein stability [11–12]. However, previous studies have shown that PEGylation has actually decreased the potency of protein therapeutics and contributed to the heterogeneity and immunoreactivity [13–15]. Fusion to the Fc-domain of IgG, or albumin, is another approach to improve proteins in circulation by interaction with neonatal Fc receptor (FcRn) [16–17]. However, the steric problems were significantly increased owing to the integration of Fc, or albumin, leading to reduced specificity. Recently, DeLano et al. identified that the IgG-Fc binding peptide (IBP) could reversibly bind with high affinity to the Fc-domain of human IgG at the interface between its C_H_2 and C_H_3 domains [18–19]. More importantly, the low molecular weight and simple structure of IBP allow better preservation of target specificity, while its non-bacterial origin leads to potentially low or no immunogenicity. Thus, IBP conjugation could be a safe and effective approach to improve the half-life of small peptides, such as HIV fusion inhibitors. However, this modification strategy of HIV-1 fusion inhibitors to extend *in vivo* half-life has not yet been reported, implicating a significant challenge in maintaining and even enhancing interaction between IBP-linked inhibitors and Fc domain of human IgG.

In the present study, we developed a novel strategy to extend the *in vivo* half-life of a short HIV-1 fusion inhibitory peptide, CP24 [20–21], by conjugating it with the human IgG Fc-binding peptide (IBP). Our results showed that IBP conjugation to the N-terminus of CP24 (IBP-CP24) exhibited more potent inhibition of HIV-1 infection than CP24 both *in vitro* and *in vivo*. Furthermore, the half-life of IBP-CP24 in the plasma of rhesus monkeys was about 26-fold longer than that of CP24. Moreover, combining IBP-CP24 with a broad neutralizing antibody (bNAb) targeting the CD4-binding site (CD4bs) in gp120 or a membrane proximal external region (MPER) in gp41 exhibited synergistic inhibitory effect on HIV-1 infection. These results suggest that IBP-CP24 has the potential to be further developed as a new HIV-1 fusion inhibitor-based, long-acting anti-HIV drug.

## Results

### Generation and characterization of IBP-conjugated peptide inhibitors

**Fig 1:**
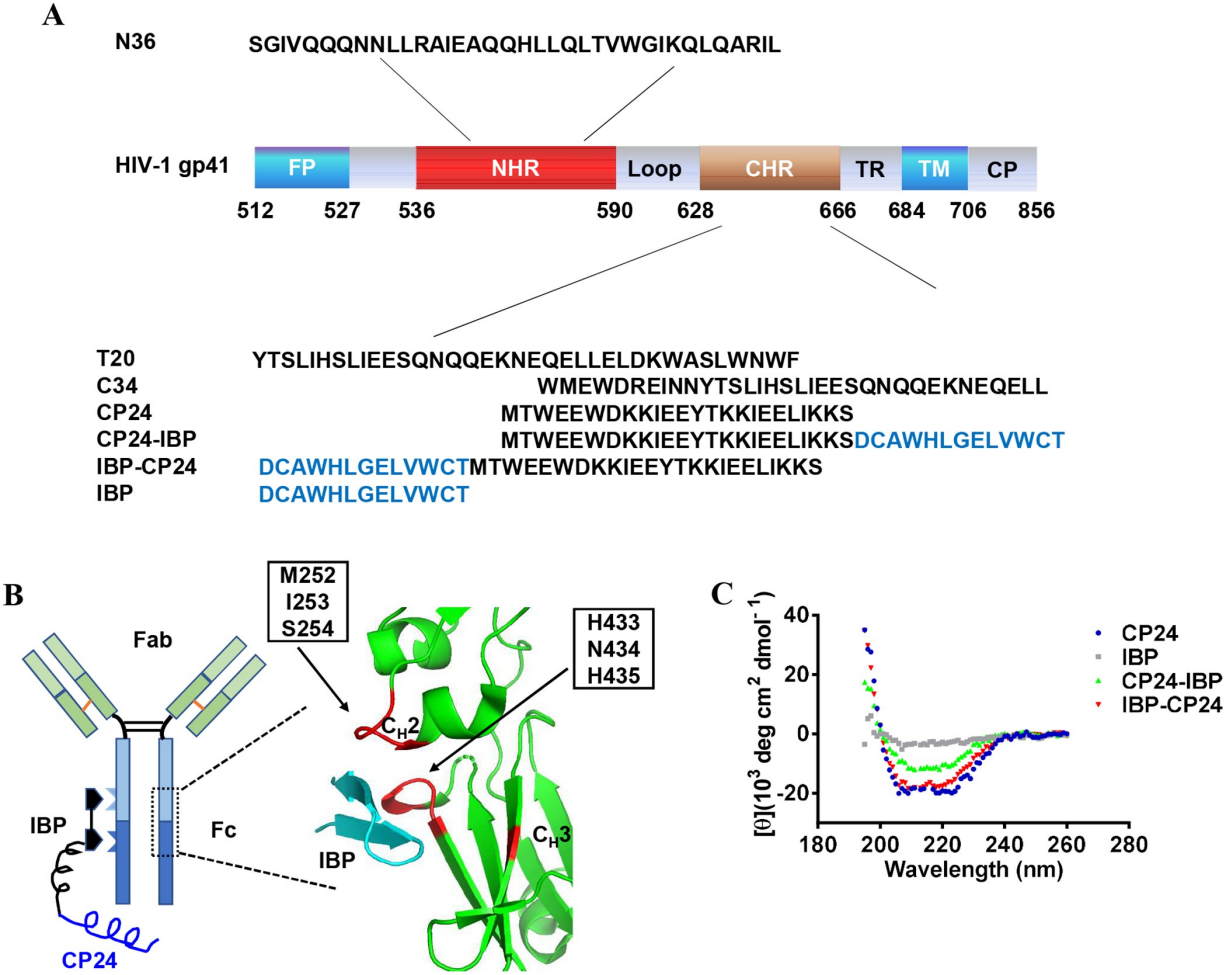
Design and characterization of IBP-conjugated peptides. (A) Schematic representation of the HIV-1 gp41 protein and sequences of peptides. FP, fusion peptide region; NHR, N-terminal heptad repeat; CHR, C-terminal heptad repeat; TR, tryptophan-rich region; TM, transmembrane region; CP, cytoplasm region. CP24 derived from CHR domain and IBP-conjugated peptides are shown in the diagram. (B) Binding sites of IBP to the Fc domain of human IgG and crystal structure of IBP in complex with human IgG Fc [18–19]. Green, Fc domain of human IgG; cyan, IBP; red, critical amino acids at CH2-CH3 interface for IBP-binding (PDB’s accession number is 1DN2). (C) Circular dichroism (CD) spectra for CP24, IBP, CP24-IBP and IBP-CP24 in phosphate buffer (pH = 7.2). The circular dichroism spectra of these inhibitors displayed typical double minima at 208 and 222 nm for the α-helical feature.

To generate a long-acting HIV fusion inhibitor, we intended to conjugate IBP to CP24, taking advantage of IBP’s ability to reversibly bind to the Fc fragment of human IgG (Fig 1A and 1B). We selected CP24 other than T20 to develop the long-acting HIV fusion inhibitor because: 1) CP24 consists of only 24 amino acids, while T20 contains 36 amino acids. It is much easier and more economic to synthesize the peptide consists of CP24 and IBP than that contains T20 and IBP (Fig 1A); and 2) CP24 is more effective than T20 against divergent HIV-1 strains, including T20-resistant strains. To determine if IBP fusion alters the original structure of CP24, we performed a circular dichroism (CD) experiment to characterize the secondary structure of peptides containing CP24 and IBP. As shown in Fig 1C, IBP-CP24, in which IBP was conjugated to the N-terminus of CP24, exhibited typical double negative peaks at 208 and 222 nm as the α-helical feature in the CD spectrum, similar to that of CP24. However, CP24-IBP, in which IBP was conjugated to the C-terminus of CP24, showed decreased α-helical contents compared with the original CP24 peptide (Fig 1C). As expected, IBP itself has no α-helical feature. This result suggests that the addition of IBP to the N-terminus of CP24 does not change the conformation of CP24.

### Addition of IBP to the N-terminus of the CP24 enhanced its anti-HIV-1 activity

**Fig 2:**
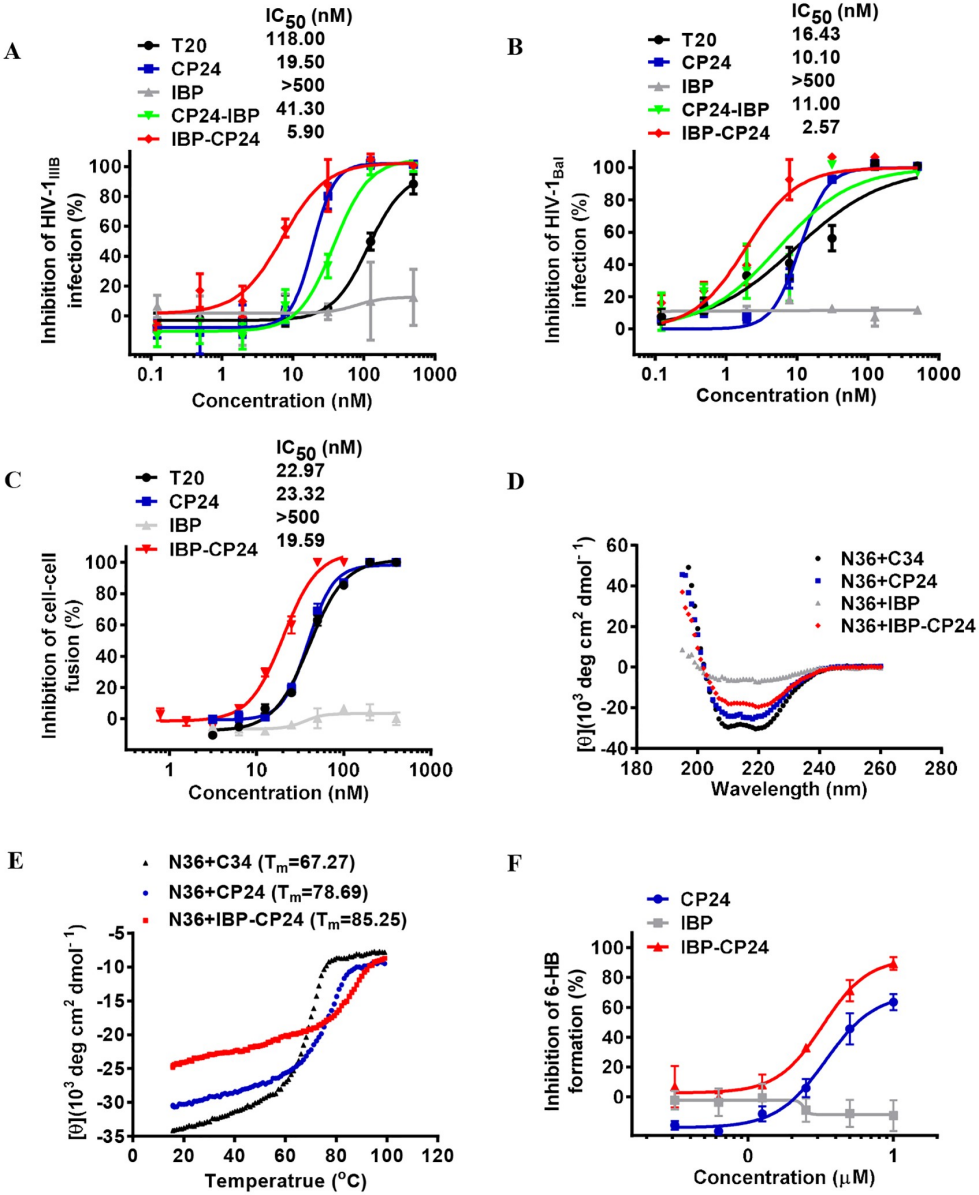
Inhibitory activities of IBP-conjugated peptides against HIV-1 infection and biophysical properties of CHR/NHR peptide complexes. (A) Inhibitory activities of T20, CP24, IBP, CP24-IBP and IBP-CP24 against infection of HIV-1 IIIB (subtype B, X4). (B) Inhibitory activities of T20, CP24, IBP, CP24-IBP and IBP-CP24 against infection of HIV-1 Bal (subtype B, R5). (C) Inhibition of Env-mediated cell-cell fusion between H9/HIV-1_IIIB_ cells and MT-2 cells. The data are presented as mean ± SD. (D) The secondary structure of the complexes of N36 and C34, CP24, IBP or IBP-CP24 was determined by CD. The circular dichroism spectra of these complexes displayed typical double minima at 208 and 222 nm for the α-helical feature. C34 and IBP were used as positive and negative controls, respectively. (E) Thermostability of the complexes of N36 with C34, CP24 or IBP-CP24 was measured by CD. (F) Inhibitory activities of CP24, IBP and IBP-CP24 against 6-HB formation between N36 and C34. Each sample was tested in triplicate and the data are presented as mean ± SD.

We next compared the antiviral activities of CP24-IBP (IBP conjugated to CP24's C-terminus) and IBP-CP24 (IBP conjugated to CP24's N-terminus) with CP24 against laboratory adapted HIV-1 strains IIIB (X4) and Bal (R5) infection in MT-2 and M7 cells. Consistent with the above result, IBP-CP24 was highly effective against HIV-1_IIIB_ and HIV-1_Bal_ infection with the 50% inhibitory concentration (IC_50_) values of 5.9 nM and 2.6 nM, respectively, about 2.3- and 2.9-fold more potent than CP24 itself (Fig 2A and 2B), while CP24-IBP showed lower inhibitory activity against HIV-1_IIIB_ and HIV-1_Bal_ infection than CP24 (Fig 2A and 2B). The results suggest that the addition of IBP to the N-terminus of CP24 can increase its anti-HIV-1 activity. Meanwhile, we performed the HIV-1 Env-mediated cell-cell fusion assay and found that IBP-CP24 could inhibit cell-cell fusion between H9/HIV-1_IIIB_ and MT-2 cells with an IC_50_ of 19.59 nM, about 1.2-fold of that of CP24 itself (Fig 2C). This result further indicates that the addition of IBP to the N-terminus of CP24 can indeed enhance its fusion inhibitory activity.

### IBP-CP24 formed stable 6-HB with N36 and blocked 6-HB formation between NHR and CHR peptides

Formation of the six-helical bundle (6-HB) core is a critical step for HIV-1 fusion and entry into target cells. Previous studies showed that CP24 could interact with the gp41 NHR peptide N36 to form 6-HB to prevent the entry of HIV into target cells [20]. To explore if IBP-CP24 utilizes the same mechanism against HIV-1 infection, we first characterized the secondary structure of the complexes of IBP-CP24 with N36 peptide by CD. IBP-CP24 and N36 were incubated at equal concentration at 37 °C for 30 min, and the CD spectra of the mixture was measured. As shown in Fig 2D, the mixture of IBP-CP24/N36 displayed typical double negative peaks at 208 and 222 nm in the CD spectrum, similar to that of CP24/N36, suggesting that the addition of IBP to the N-terminus of CP24 does not significantly alter the function of CP24.

We then compared the thermostability of the 6-HBs formed between CP24 or IBP-CP24 and N36. As shown in Fig 2E, the T_m_ value of the 6-HB formed between N36 and IBP-CP24 (85.25 °C) was higher than that of the 6-HB formed between N36 and C34 (67.27 °C) or CP24 (78.69 °C), suggesting that the binding affinity between N36 and IBP-CP24 was much higher than that between N36 and C34 or CP24. This result indicates that IBP-CP24 can form more stable heterogeneous 6-HBs with viral gp41 NHR than those formed between C34 or CP24 and viral gp41 NHR.

We next used a sandwich ELISA to determine if IBP-CP24 would show inhibitory activity against 6-HB formation of viral gp41. As shown in Fig 2F, IBP-CP24 exhibited much stronger inhibition on 6-HB formation than CP24 itself, indicating that the addition of IBP to the N-terminus of CP24 could interact with gp41 NHR and block 6-HB formation between viral gp41 NHR and CHR.

All the above results suggest that, compared with CP24 itself, IBP-CP24 binds to gp41 NHR to form more stable heterogeneous 6-HB, thus being more effective in blocking homologous 6-HB formation between the viral gp41 CHR and NHR and in inhibiting viral fusion with and entry into the target cell.

### IBP-CP24 could bind to human and rhesus monkey IgG and showed longer half-life *in vivo*

**Fig 3:**
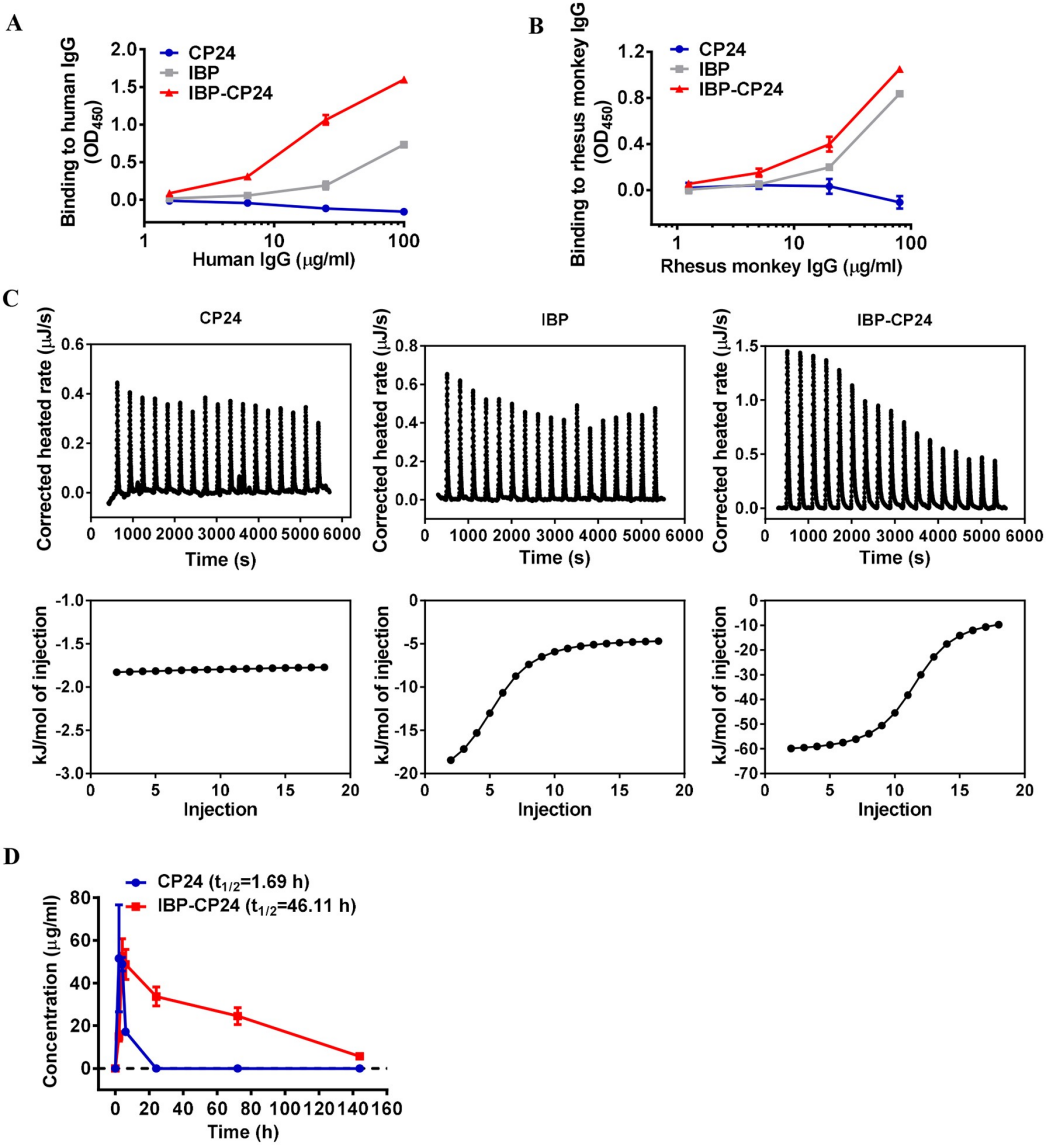
*In vitro* human and rhesus monkey IgG binding and *in vivo* pharmacokinetic studies of CP24 and IBP-CP24. (A) Binding affinity of CP24, IBP and IBP-CP24 to human IgG was determined by ELISA. (B) Binding affinity of CP24, IBP and IBP-CP24 to rhesus monkey IgG was measured by ELISA. Each sample was tested in triplicate and the data are presented as mean ± SD. (C) Binding affinity of CP24, IBP and IBP-CP24 to rhesus monkey IgG was evaluated by isothermal titration calorimetry (ITC) assay. The top panel showed the titration traces inhibitors solution injected into rhesus monkey IgG solution. The bottom panel showed the binding affinity of inhibitors to rhesus monkey IgG. (D) Plasma concentrations of CP24 and IBP-CP24 *in vivo*. Rhesus monkeys were administered intravenously with a single dose of 10 mg/kg CP24 (n = 2) or IBP-CP24 (n = 3). The concentrations were determined by sandwich ELISA. Each sample was tested in triplicate and data were presented as mean ± SD.

Previous studies have shown that IBP could bind specifically with human IgG Fc [18]. We investigated whether conjugation of IBP with CP24 could affect the binding affinity of IBP to IgG Fc by using ELISA. The result showed that although both IBP and IBP-CP24 could bind to human IgG in a dose-dependent manner, IBP-CP24 exhibited higher binding affinity with human IgG than IBP alone, while CP24 alone could not bind with human IgG (Fig 3A). We found that the IBP binding sites on human IgG Fc have similar sequence as those on rhesus monkey IgG by sequence comparison, we thus planned to use rhesus monkey to evaluate the *in vivo* stability of CP24 and IBP-CP24. We first compared the monkey IgG-binding activity of IBP-CP24 with that of IBP. As shown in Fig 3B and S1 Table, IBP-CP24 exhibited higher binding affinity to rhesus monkey IgG and human IgG than IBP itself (Fig 3B), while the binding affinity between IBP-CP24 and rhesus monkey IgG (Kd = 5.57E-07 M) was about 2-fold lower than between IBP-CP24 and human IgG (Kd = 1.77E-07 M), which is consistent with the previous reports [18].

We then compared the binding isotherms and binding affinity of monkey IgG with CP24, IBP or IBP-CP24, using isothermal titration calorimetry (ITC). As shown in Fig 3C and Table 1, all reactions were driven by the enthalpy change (ΔH < 0). While CP24 itself had low, or no, binding affinity to monkey IgG, IBP-CP24 bound with monkey IgG with a Kd of 9.46E-07 M, about 1.6-fold more potent than that of IBP (2.44E-06 M). These results suggest that linking IBP to the N-terminus of CP24 does not reduce, but rather enhances IBP binding with human or rhesus monkey IgG (Fig 3A–3C).

**Table 1:** Thermodynamic parameters of CP24, IBP or IBP-CP24 binding to rhesus monkey IgG, as determined by ITC.

Complex	Kd(M)	ΔH(KJ/mol)	ΔS(J/mol.K)
**CP24/rhesus monkey IgG**	1.00E-03	-25.61	-28.46
**IBP/rhesus monkey IgG**	2.44E-06	-16.90	50.79
**IBP-CP24/rhesus monkey IgG**	9.46E-07	-54.23	-66.54

To evaluate the pharmacokinetics of CP24 and IBP-CP24 *in vivo*, we administered a single dose of CP24 or IBP-CP24 to rhesus monkeys intravenously (i.v.) and collected plasma at different time points. The plasma concentrations of CP24 and IBP-CP24 were determined by sandwich ELISA. In rhesus monkeys receiving 10 mg/kg IBP-CP24, the mean half-life (t_1/2_) was about 46 h (Fig 3D and Table 2), 26-fold longer than that of CP24 (t_1/2_ = 1.69 h), and about 14-fold longer than the reported t_1/2_ (2.5~3.5 h) of T20 [22]. The mean area under the concentration-time curve (AUC 0-t) of IBP-CP24 was 3,429 μg/mL*h, about 18-fold higher than that of CP24 (180 μg/mL*h) (Table 2). We also used an assay for measuring the *in vitro* IC_50_ and *ex vivo* anti-HIV-1 activity of CP24 and IBP-CP24, based on which the concentration of the active peptides in the plasma samples was estimated and their half-life was calculated [23–26]. As shown in S1 Fig and S2 Table, the half-life of IBP-CP24 and CP24 in the plasma of rhesus monkeys was about 44.8 h and 1.4 h, respectively, which is consistent with the result obtained from the sandwich ELISA.

**Table 2:** Pharmacokinetic parameters of CP24 (n = 2) and IBP-CP24 (n = 3) in rhesus monkeys.

Parameter	CP24	IBP-CP24
t_1/2_ (h)	1.69	46.11
Tmax (h)	2	4
Cmax (μg/mL)	23.56	52.67
AUC 0-t (μg/mL*h)	180.10	3429.16
MRT 0-inf_obs (h)	4.47	64.41

### IBP-CP24 exhibited broad and potent inhibitory activity against HIV-1 clinical strains, pseudotyped viruses and T20-resistant strains

To study the spectrum of IBP-CP24 on different HIV stains, we evaluated the antiviral activities of IBP, T20, CP24 and IBP-CP24 against a panel of HIV-1 clinical strains of different subtypes (A, B, C, D, F, O and A/E) and coreceptors (X4, R5 and X4/R5), as well as pseudotyped viruses with a panel of HIV-1 Env. As shown in Table 3, IBP could not inhibit the infection by any HIV-1 clinical strains or pseudoviruses at concentration as high as 1,000 nM, while IBP-CP24 could effectively inhibit infection by 8 HIV-1 clinical strains tested in a dose-dependent manner with an average IC_50_ of 32.3 nM, about 2.5- and 1.1-fold of that of T20 and CP24, respectively. Similarly, IBP-CP24 could also efficiently suppress infection by 12 pseudotyped HIV-1 strains tested with an average IC_50_ of 13.3 nM, about 24.2-fold and 4.9-fold more potent than T20 and CP24, respectively. These results suggest that the addition of IBP to CP24 did not decrease but increase its anti-HIV-1 activity.

**Table 3:** Inhibitory activity of IBP, T20, CP24, and IBP-CP24 against HIV-1 clinical strains and pseudovirus.

Virus	Subtype	Coreceptor usage	IC_50_ (nM)			
			IBP	T20	CP24	IBP-CP24
**HIV-1 clinical strains**						
92UG029	A	X4	>1000	22.4±31.6	0.8±0.2	0.7±0.3
HIV-1 US4	B	R5	>1000	42.2±54.3	29.8±37.6	14.8±5.3
HIV-1 TZA68/125A	C	R5	>1000	>500	151.2±52.5	173.7±54.7
92UG024	D	X4	>1000	9.0±6.8	6.9±0.3	26.2±24.8
HIV-1 J32228M4	D	R5	>1000	12.9±16.8	36.8±7.6	5.9±1.6
93/BR/020	F	X4/R5	>1000	36.3±25.5	7.9±4.1	21.3±17.7
BCF02	O	R5	>1000	0.5±0.2	8.2±7.7	4.4±0.5
92TH009	A/E	R5	>1000	13.0±25.5	32.7±13.6	11.0±4.8
Average			>1000	79.5	34.3	32.3
**Pseudoviruses**						
SC422661.8	B	R5	>1000	26.6±19.4	31.7±20.1	0.2±0.3
ZM109F.PB4	C	R5	>1000	>500	1.0±0.8	11.4±2.2
AE03	A/E	R5	>1000	356.9±130.3	21.2±15.7	24.5±8.0
GX11.13	A/E	R5	>1000	277.6±189.2	279.5±93.7	29.6±15.9
GX2010.36	A/E	R5	>1000	268.9±300.9	170.8±96.4	33.2±17.7
SHX335.24	A/E	R5	>1000	>500	2.6±2.9	8.1±4.6
CRF01_AE clone 269	A/E	R5	>1000	>500	55.6±23.7	6.3±3.0
HB5–3	B/C	R5	>1000	46.3±23.9	9.1±4.1	11.3±2.2
BC02	B/C	R5	>1000	>500	5.6±1.8	2.9±0.8
SC19–15	B/C	R5	>1000	>500	0.7±0.6	0.7±0.1
CH119	B/C	R5	>1000	>500	3.8±3.8	12.7±4.6
43–22	B’	R5	>1000	55.0±18.2	361.5±237.5	19.0±8.0
Average			>1000	335.9	78.6	13.3

Each sample was tested in triplicate and data was presented in mean ± SD. When the average was calculated, the IC_50_ value of >500 nM was treated as 500 nM.

We next assessed the antiviral activity of IBP, T20, CP24 and IBP-CP24 against HIV-1 variants with single or double mutations in gp41 that are resistant to the first HIV fusion inhibitor T20 [27]. As shown in Table 4, IBP and T20 were unable to inhibit any T20-resistant strains at concentrations as high as 1,000 nM and 500 nM, respectively, while IBP-CP24 was highly effective in inhibiting infection by T20-resistant HIV-1 strains with an average IC_50_ of 21.9 nM, about 1.5-fold of that of CP24. These results indicate that both IBP-CP24 and CP24 are highly effective against T20-resistant strains.

**Table 4:** Inhibitory activity of IBP, T20, CP24, and IBP-CP24 against T20-resistant strains.

T20-resistant HIV-1 variants	IC_50_ (nM)			
	IBP	T20	CP24	IBP-CP24
V38A	>1000	>500	38.1±9.2	49.2±7.6
V38E N42S	>1000	>500	30.8±16.0	9.2±3.0
V38A N42T	>1000	>500	9.8±3.0	3.6±1.2
D36G	>1000	>500	50.6±24.3	31.8±9.1
V38A N42T	>1000	>500	35.5±25.1	15.9±5.2
Average	>1000	>500	33.0	21.9

Each sample was tested in triplicate and data was presented in mean ± SD.

### IBP-CP24 showed no *in vitro* or *in vivo* toxicity

**Fig 4:**
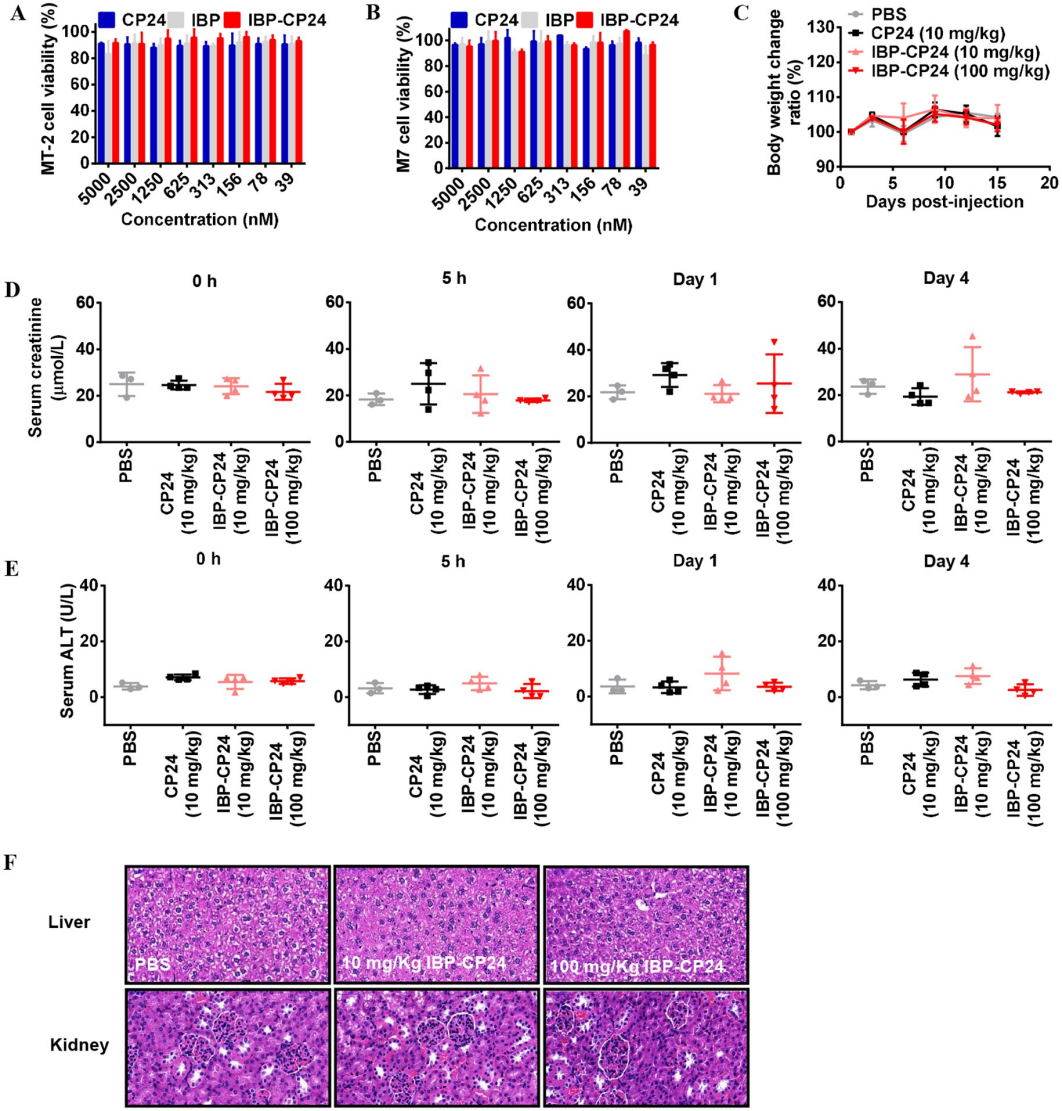
Evaluation of *in vitro* and *in vivo* toxicity of IBP-CP24. (A and B) Cytotoxicity of CP24, IBP and IBP-CP24 to MT-2 (A) and M7 (B) cells. Each sample was tested in triplicate and the data are presented as mean ± SD. (C) Body weight changes were recorded at the indicated time points in mice treated with PBS, 10 mg/kg CP24, 10 mg/kg IBP-CP24 or 100 mg/kg IBP-CP24. Each time point is represented by mean ± SD. (D) The creatinine in mice sera was measured by using a creatinine assay kit at different time points. Error bars indicate standard deviations. (E) ALT activity in the sera of mice was measured with an ALT assay kit. Error bars indicate standard deviations. (F) H&E staining analysis of livers and kidneys in treated mice at day 15 post-injection.

It was reported that long-term use of antiretroviral therapy may cause histopathological and histomorphological changes in the liver and kidneys [28–29], and the elevated serum creatinine and alanine aminotransferase (ALT) [30]. Therefore, it is necessary to evaluate the potential *in vitro* and *in vivo* toxicity of IBP-CP24. To study the *in vitro* cytotoxicity of IBP-CP24, MT-2 and M7 cells used for testing of inhibitory activity of peptides on HIV-1 infection were treated with a graded concentration of CP24, IBP, or IBP-CP24 for 48 h before evaluation of their viability with CCK-8. As shown in Fig 4A and 4B, IBP-CP24 showed no significant *in vitro* cytotoxicity to MT-2 and M7 cells at the concentration as high as 5 μM, more than 800-fold higher than its IC_50_ for HIV-1 inhibition. Similarly, both IBP and CP24 at 5 μM showed no *in vitro* cytotoxicity to MT-2 and M7 cells (Fig 4A and 4B).

To investigate the potential *in vivo* toxicity of IBP-CP24, ICR mice were administered with PBS, 10 mg/kg CP24 in PBS, 10 mg/kg IBP-CP24 in PBS, or 100 mg/kg IBP-CP24 in PBS. Their behavior and body weight were recorded for two weeks after peptide administration. Our results showed that mice treated with low dose (10 mg/kg) and high dose (100 mg/kg) of IBP-CP24 lived normally without any sign of discomfort and showed similar body weight change as that of the mice in the PBS- and CP24-treatment groups (Fig 4C). We further measured the levels of creatinine and alanine aminotransferase (ALT) in the sera of the peptide-treated mice. There was no significant difference on the levels of creatinine (Fig 4D) and ALT (Fig 4E) at all time points among the mice treated with PBS, 10 mg/kg CP24, 10 mg/kg IBP-CP24, or 100 mg/kg IBP-CP24, indicating that the administration of IBP-CP24 at low or high dose had no impact on the renal and hepatic functions of mice. The hematoxylin and eosin (H&E) stained sections of liver and kidneys from IBP-CP24-treated mice showed no pathological abnormality compared with PBS-treated mice (Fig 4F). These results suggest that like PBS and CP24, IBP-CP24 has no obvious *in vivo* toxicity to the treated mice.

### IBP-CP24 treatment protected humanized mice from HIV-1 infection

To evaluate the *in vivo* antiviral efficacy of IBP-CP24 and CP24 against HIV-1 JR-CSF infection in humanized mice, we first evaluated the *in vitro* inhibitory activity of IBP-CP24 and CP24 against infection of HIV-1 JR-CSF (a CCR5-tropic strain commonly used for infection of humanized mice) in TZM-bl cells. We demonstrated that both IBP-CP24 and CP24 could effectively inhibit HIV-1 JR-CSF infection *in vitro* in a dose-dependent manner with IC_50_ of 6.3 and 13.9 nM, respectively (S2 Fig).

**Fig 5:**
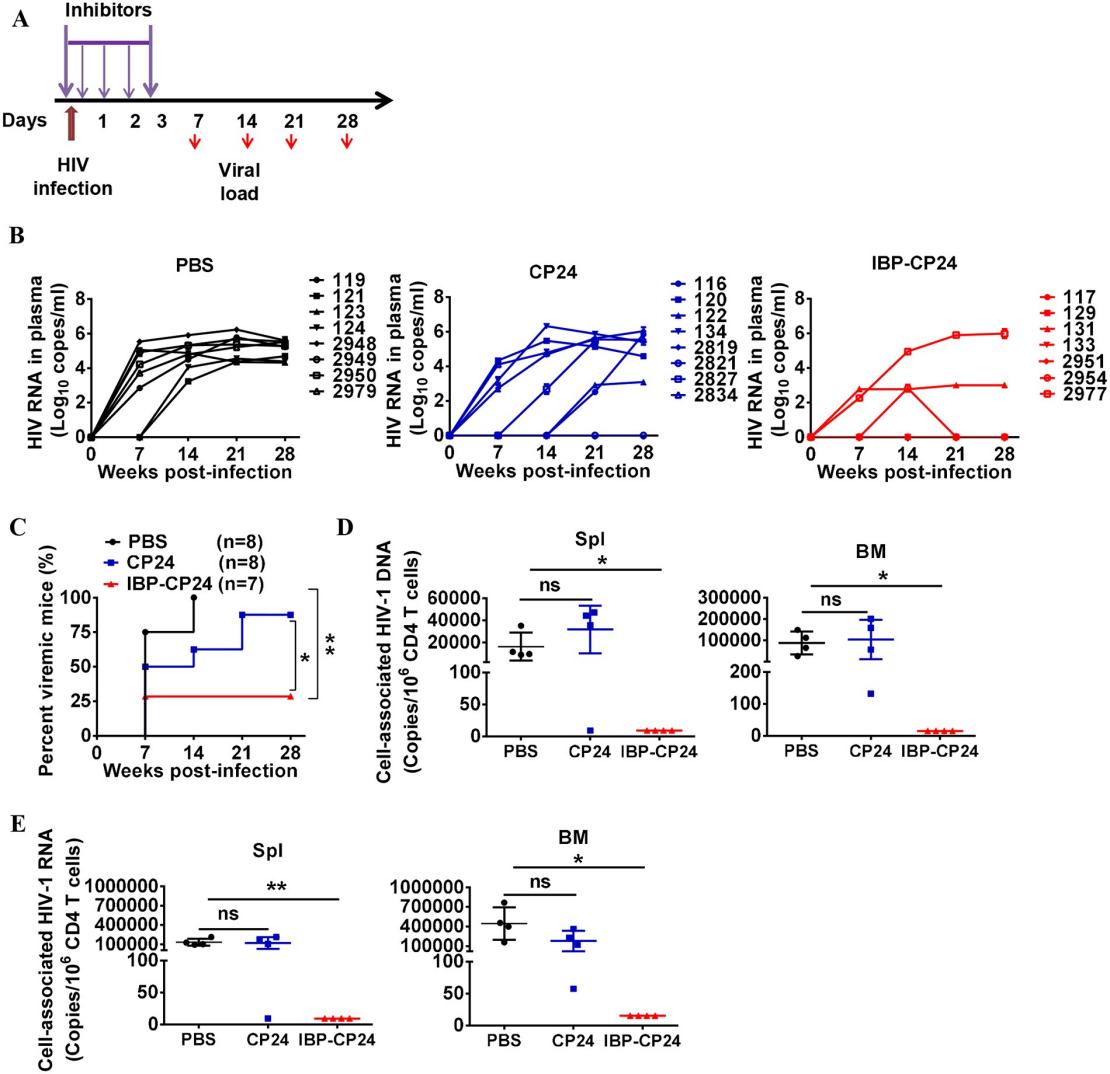
*In vivo* evaluation of the prophylactic activity of CP24 and IBP-CP24 in humanized mice. (A) Schematic diagram of the prophylactic experimental design. Humanized mice were challenged with HIV-1 JRCSF at 2 h post-treatment with PBS, CP24 and IBP-CP24. Mice were then injected with PBS, CP24 and IBP-CP24 peptides once at 6 h post-infection and then once every day for 3 consecutive days. (B) Plasma HIV-1 RNA levels were monitored at indicated time points in mice treated with PBS, CP24 or IBP-CP24. Shown are combined viremic data of two independent experiments. Each line represents a single mouse. (C) Summary of prophylactic protection of CP24 and IBP-CP24. Shown are summarized data of 2 independent experiments. PBS, n = 8; CP24, n = 8; IBP-CP24, n = 7. (D and E) Cell-associated HIV-1 DNA (D) and RNA (E) in human cells from mouse spleen and bone marrow were quantified by real-time PCR. All mice were terminated at day 28 post-infection. Shown are representative data of one independent experiment. Data were presented as mean ± SD. Log-rank test (C) or unpaired, 2-tailed *t* test (D and E) was performed. *P<0.05, ** P<0.01.

We then investigated the protective efficacy of IBP-CP24 and CP24 against HIV-1 JR-CSF infection in humanized Nod-rag-gC (NRG-hu HSC) mice. Humanized mice were challenged with HIV-1 JR-CSF at 2 h post-treatment with PBS, CP24 or IBP-CP24 (Fig 5A). At 7 days post-infection (dpi), 75% of mice treated with PBS became HIV-1 viremia-positive with a high level of viral load (about 4.4 log_10_ copies/mL), while 50% of mice treated with CP24 were HIV-1 viremia-positive with a middle level of viral load (about 3.6 log_10_ copies/mL). In contrast, only 28.5% of the mice treated with IBP-CP24 exhibited HIV-1 viremia-positive with a low level of viral load (about 2.5 log_10_ copies/mL). At 14 dpi, 100% and 62.5% of the mice treated with PBS and CP24, respectively, showed a high level of viremia, while only 28.5% of mice treated with IBP-CP24 showed detectable viremia (Fig 5B and 5C). We further evaluated the levels of cell-associated HIV-1 DNA and RNA in the spleen and bone marrow of the treated mice at day 28 post-infection. As shown in Fig 5D and 5E, undetectable cell-associated HIV-1 DNA and RNA were found in the spleen and bone marrow of the mice treated with IBP-CP24, while high levels of cell-associated HIV-1 DNA and RNA were detected in the spleen and bone marrow of the mice treated with PBS and CP24. These results suggest that IBP-CP24 is much more potent than CP24 in preventing HIV-1 infection in humanized mice. Thus, IBP-CP24 has potential to be further developed as a prophylactic agent to prevent HIV-1 infection in high-risk populations.

### IBP-CP24 treatment inhibited HIV-1 replication during persistent infection *in vivo* when co-administered with normal human IgG

**Fig 6:**
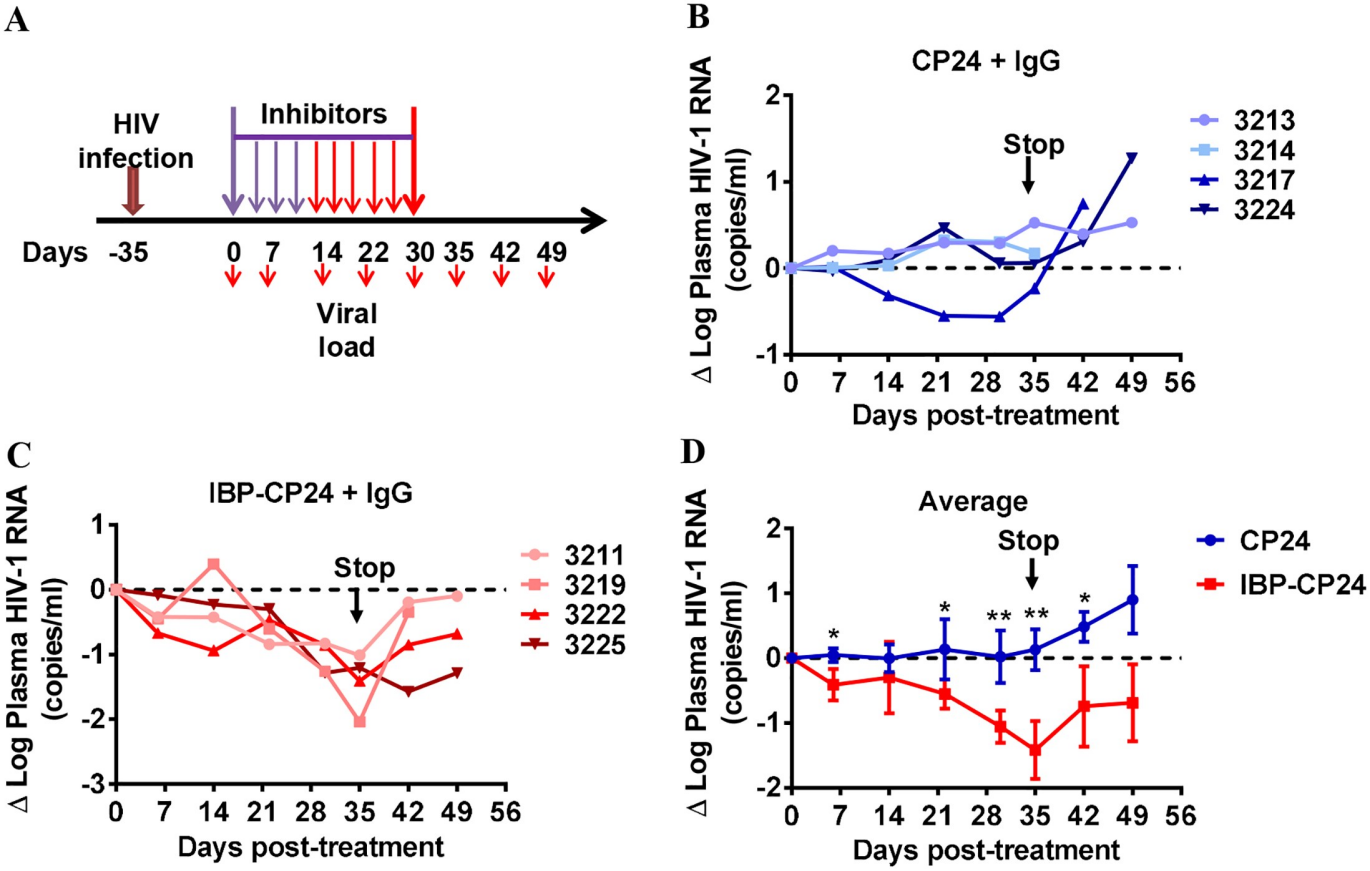
*In vivo* evaluation of the therapeutic efficacy of CP24 and IBP-CP24 in humanized mice with established HIV-1 infection. (A) Schematic diagram of the therapeutic experimental design. Humanized mice were infected with HIV-1 at day -35. At day 0, the infected mice received CP24 or IBP-CP24 treatment daily through day 14 (purple arrows). Beginning from day 14 to day 35, the mice received CP24 or IBP-CP24 treatment twice a day (red arrows). All mice were injected with human IgG twice every week. (B and C) The change of plasma viremia from baseline was monitored at the indicated time points in mice treated with CP24 (B) and IBP-CP24 (C). (D) Comparison of the changes of plasma HIV-1 RNA in humanized mice treated with CP24 or IBP-CP24. Each sample was tested in triplicate and data were presented as mean ± SD. (CP24 + IgG, n = 4; IBP-CP24 + IgG, n = 4). Unpaired, 2-tailed *t* test was performed. *P<0.05, ** P<0.01.

We further assessed the therapeutic efficacy of IBP-CP24 in humanized mice with persistent HIV-1 infection (Fig 6A and S3A Fig). Due to the absence of human IgG in humanized NRG mice [31], we investigated whether co-injection of IBP-CP24 with normal human IgG could improve its therapeutic efficacy in HIV-1-infected humanized mice. Coinjection of IBP-CP24 with human IgG once daily for 14 days showed minimal effect on plasma HIV-1 RNA levels (mean Δlog_10_ plasma HIV-1 RNA: 0.13 log_10_; Fig 6C and 6D), possibly because the concentration of the peptides was not high enough to inhibit virus infection. In order to increase the concentration of the peptides in the treated mice, we switched the treatment stratagem to twice daily beginning from day 14 post-treatment. We found that co-administration of IBP-CP24 with normal human IgG resulted in gradual decrease of the plasma HIV-1 RNA (Fig 6C and S3C Fig). After twice daily treatment with IBP-CP24 plus human IgG for 21 days (i.e., day 35 post-treatment), the plasma HIV-1 RNA was significantly decreased by an average of 1.42 log_10_ (Fig 6C and 6D). As expected, HIV-1 rebounded in all humanized mice after IBP-CP24 cessation at 35 days. We used similar treatment stratagem to treat mice with CP24 and noticed that the plasma HIV-1 RNA in only one of the four mice was decreased and the average viral load in all four mice at each time point was significantly higher than that in the mice treated with IBP-CP24 (Fig 6B–6D; S3B Fig). Here, we speculate that CP24, due to a short half-life (1.69 h), might require more frequent treatment to achieve efficacy. These findings suggest that the human IgG could help stabilize IBP-CP24 *in vivo* and enhance its therapeutic activity. Therefore, IBP-CP24 could be potentially developed as a long-acting HIV fusion inhibitor for use in humans.

### IBP-CP24 intravenously administered did not induce significant IBP-CP24-specific antibody response in rhesus monkeys

**Fig 7:**
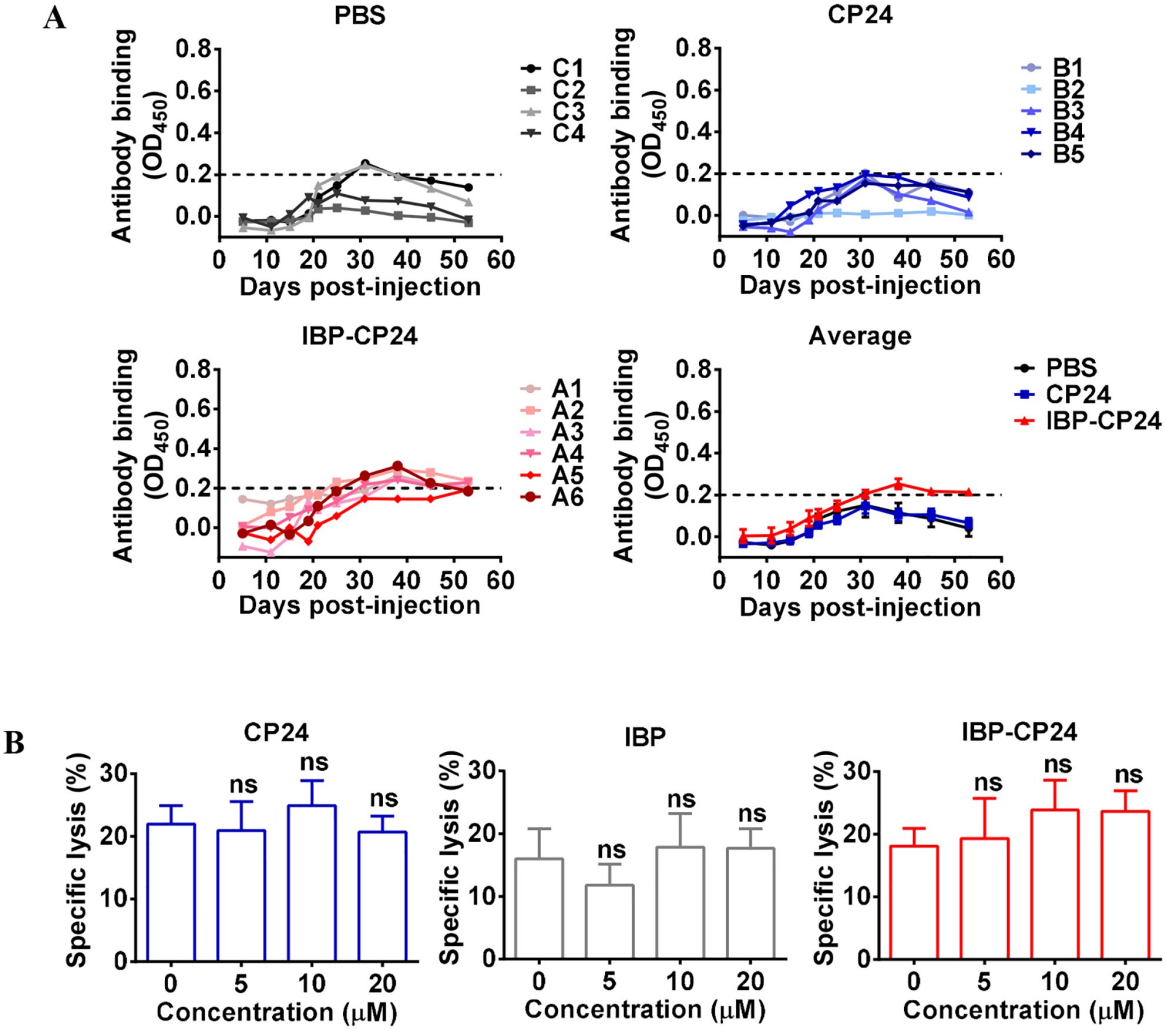
Detection of CP24 or IBP-CP24-specific antibody in the plasma of rhesus monkeys intravenously administered with CP24 or IBP-CP24 by indirect ELISA and effect of the peptides on antibody-mediated ADCC. (A) CP24 or IBP-CP24-specific antibody response in the CP24- or IBP-CP24-treated rhesus monkeys. The level of CP24 or IBP-CP24-specific IgG in the plasma of rhesus monkeys intravenously administered with PBS, CP24 or IBP-CP24 by indirect ELISA. The data were presented as mean ± SD. (B) Specific lysis of H9/HIV-1_IIIB_-infected cells mediated by 10E8 and PBMC via antibody-dependent cell-mediated cytotoxicity (ADCC). Each sample was tested in triplicate and data were presented as mean ± SD. Unpaired, 2-tailed *t* test was performed. ns, no significance.

To investigate whether the *in vivo* application of IBP-CP24 can induce IBP-CP24-specific antibody, three groups of rhesus monkeys were intravenously administered with PBS, CP24 (10 mg/kg), and IBP-CP24 (10 mg/kg), respectively, daily for one month. The plasma samples were collected at the indicated time points for detecting CP24 or IBP-CP24-specific antibody using an indirect ELISA. As shown in Fig 7A, there was no significant detectable antibodies specific for CP24 or IBP-CP24 in the sera of rhesus monkeys at different time points within two months after the treatment with PBS, CP24 or IBP-CP24, respectively, started. We then adapted a captured peptide ELISA, in which streptavidin was immobilizing for capture of the biotinylated CP24 or IBP-CP24 peptides, to detect specific antibodies specific for CP24 or IBP-CP24 and found that there was no significant difference in the levels of the specific antibodies in the plasma between the PBS-treated monkeys and CP24- or IBP-CP24-treated monkeys (S4 Fig), which is fully consistent with the results obtained from the indirect ELISA. These findings suggest that intravenously injected CP24 or IBP-CP24 in the absence of adjuvant could not induce significant antibody responses against CP24 or IBP-CP24 in rhesus monkeys.

### IBP-CP24 did not affect antibody-mediated antibody-dependent cellular cytotoxicity (ADCC)

One of the most relevant functions mediated by IgG Fc is ADCC through Fc binding with the Fc receptors (FcRs) on effector cells, including natural killer (NK) cells or monocytes [32]. We thus evaluated the impact of IBP on human IgG-mediated ADCC. The 10E8 monoclonal antibody that targets the HIV-1 gp41 membrane proximal external region (MPER) can mediate potent ADCC [33–34]. Therefore, we assessed the effect of IBP on 10E8 antibody-mediated ADCC by using PBMCs as effector cells (EC) and H9/HIV-1_IIIB_ as target cells (TC). As shown in Fig 7B, 10E8-mediated specific lysis was about 20%, and lytic activity remained about the same with the addition of CP24, IBP or IBP-CP24 at the concentration as high as 20 μM. These results suggest that the presence of IBP-CP24 did not interfere with antibody 10E8-mediated specific lysis of HIV-infected cells and that IBP-CP24 could be potentially used in combination with a bNAb.

### Combination of IBP-CP24 and a bNAb targeting the site in the HIV-1 Env different from that for IBP-CP24 exhibited synergistic effect against HIV-1 infection

**Fig 8:**
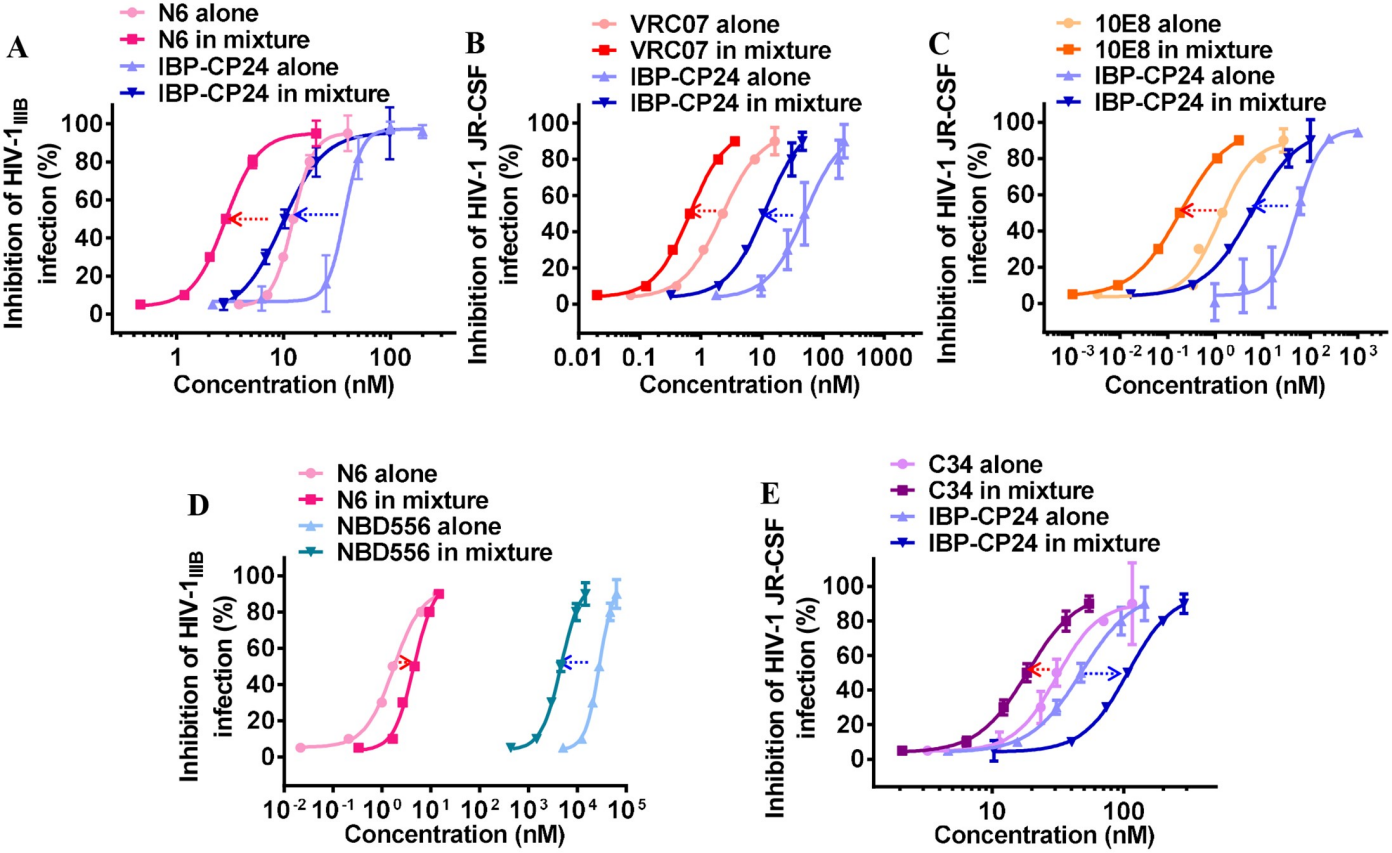
Synergistic effect of IBP-CP24 combined with bNAbs against HIV-1 infection *in vitro*. (A) The inhibitory activities of N6 alone, IBP-CP24 alone, or N6/IBP-CP24 combination against HIV-1 IIIB infection. (B) The inhibitory activities of VRC07 alone, IBP-CP24 alone, or VRC07/IBP-CP24 combination against HIV-1 JR-CSF infection. (C) The inhibitory activities of 10E8 alone, IBP-CP24 alone, or 10E8/IBP-CP24 combination against HIV-1 JR-CSF infection. (D) The inhibitory activities of N6 alone, NBD556 alone, or N6/NBD556 combination against HIV-1 IIIB infection. (E) The inhibitory activities of C34 alone, IBP-CP24 alone, or C34/IBP-CP24 combination against HIV-1 JR-CSF infection. Each sample was tested in triplicate and data were presented as mean ± SD.

Subsequently, we evaluated the combination of the gp41-targeting HIV-1 fusion inhibitor IBP-CP24 and the gp120-targeting bNAb N6 at a molar ratio of 6: 1 (starting concentration of IBP-CP24 and N6 is 171.5 nM and 28.5 nM, respectively) based on their IC_50_ values for potential synergism against HIV-1 infection. The anti-HIV-1 activity of N6 alone, IBP-CP24 alone and N6/IBP-CP24 combination was detected respectively and the combination index (CI) and dose-reduction of the inhibitors in combination were analyzed using CalcuSyn software kindly provided by Dr. T. C. Chou [35–36]. As shown in Fig 8A and Table 5, combining IBP-CP24 with N6 exhibited a significant synergistic effect against HIV-1_IIIB_ infection with a CI of 0.58, with dose reduction of 3.6-fold for IBP-CP24 and 3.3-fold for N6, respectively. This result suggests that the synergistic anti-HIV-1 effect of the IBP-CP24/N6 combination is because they target different sites in the HIV-1 Env, i.e., the peptide IBP-CP24 targets the NHR of the HIV-1 gp41, while the bNAb N6 targets the CD4bs in the HIV-1 gp120 [37]. To confirm this hypothesis, we tested the combinations of IBP-CP24 with the bNAb VRC07 that also targets the CD4bs in gp120 [38] and the bNAb 10E8 that targets the membrane-proximal external region (MPER) in gp41 [39]. As shown in Fig 8B and 8C and Table 5, the IBP-CP24/VRC07 and IBP-CP24/10E8 combinations exhibited synergistic and strong synergistic effect against HIV-1 JR-CSF infection with CI of 0.54 and 0.17, respectively. These results confirm that the combination of the two inhibitors with different target sites exhibits synergistic effect. We also tested the combination of the bNAb N6 and NBD556, a small molecule HIV-1 entry inhibitor targeting the CD4bs in gp120 [40], which is the same target site for N6, and the combination of IBP-CP24 and C34, which is a peptide-based HIV-1 entry inhibitor targeting the NHR region of gp41, the same target site for IBP-CP24 [41]. As shown in Fig 8D and 8E and Table 5, the N6/NBD556 and IBP-CP24/C34 combinations showed antagonistic effect against HIV-1 infection with CI of 2.50 and 2.37, respectively, confirming that the combination of the two inhibitors with same target sites generally has no synergistic, but antagonistic effect.

**Table 5:** Inhibitory activity of IBP-CP24 (C34 as a control) or bNAbs (NBD556 as a control) when used alone or in combination against infection HIV-1 IIIB or JR-CSF^a^.

Inhibitor A	CI	IC_50_ (nM)		Dose reduction (fold)	Inhibitor B	IC_50_ (nM)		Dose reduction (fold)
		Alone	In mixture			Alone	In mixture	
N6	0.58	6.39	1.92	3.3	IBP-CP24	41.42	11.52	3.6
VRC07	0.54	2.22	0.66	3.4	IBP-CP24	50.54	11.55	4.4
10E8	0.17	1.26	0.19	6.6	IBP-CP24	52.67	5.82	9.0
N6	2.50	1.74	4.59	0.4	NBD556	28050	4610	6.1
C34	2.37	30.94	18.51	1.7	IBP-CP24	47.79	108.2	0.4

^a^The combination effect was analyzed by using the CalcuSyn software [36]. CI values of <0.95 and >1.05 indicate synergism and antagonism, respectively [35].

**Fig 9:**
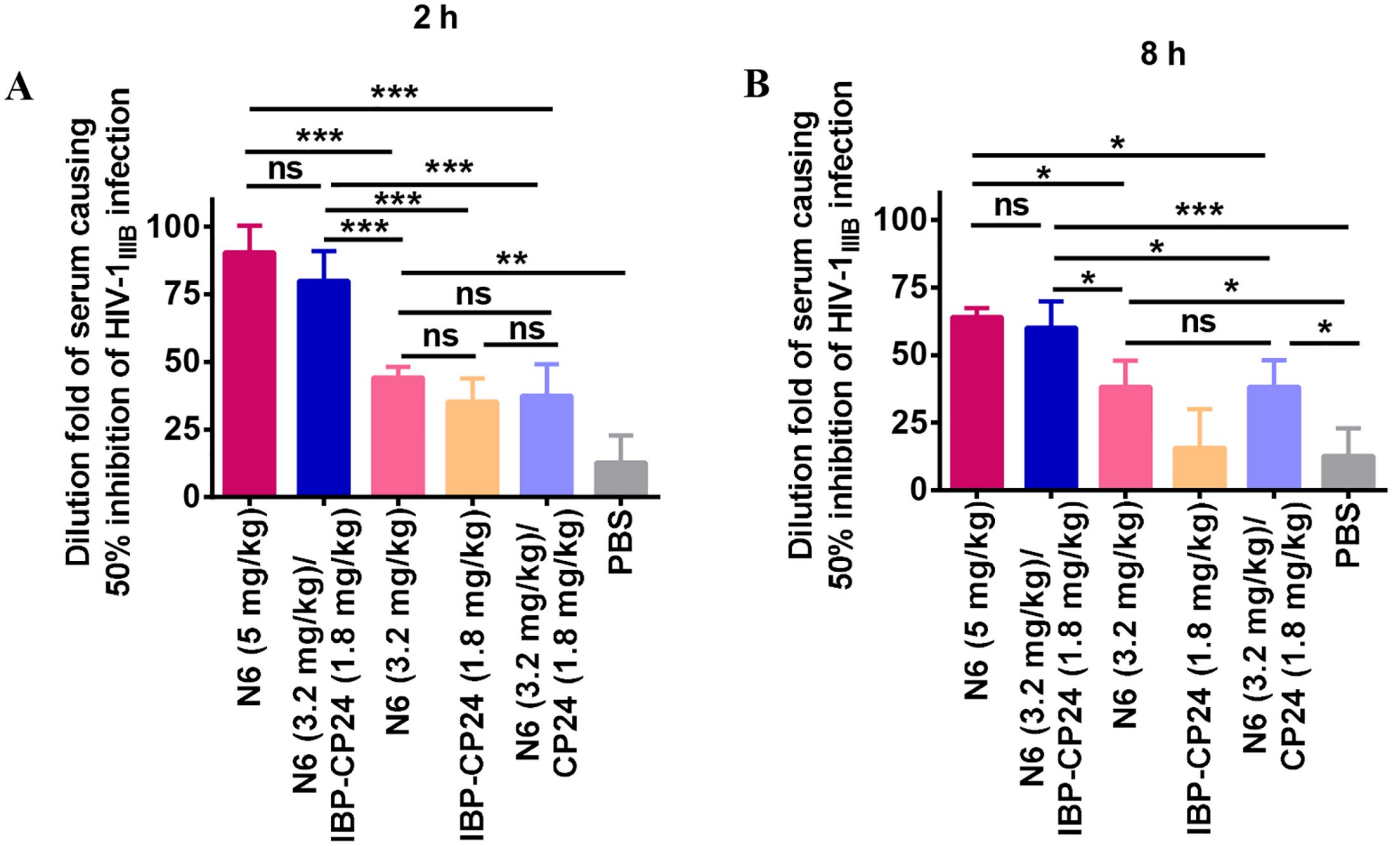
The *ex vivo* anti-HIV-1 activity of IBP-CP24 in combination with N6 at different concentration. The anti-HIV-1 IIIB activity of the sera collected from the mice 2 h (A) and 8 h (B) post-injection via *i*.*p*. with the N6 (5 mg/kg), N6 (3.2 mg/kg)/IBP-CP24 (1.8 mg/kg) combination, N6 (3.2 mg/kg), IBP-CP24 (1.8 mg/kg), and N6 (3.2 mg/kg)/CP24 (1.8 mg/kg) combination, respectively. The dilution fold of the serum causing 50% inhibition of HIV-1 IIIB infection (DF-IC50) was calculated. Each sample was tested in triplicate and data were presented as mean ± SD. Unpaired, 2-tailed *t* test was performed. "ns" means no significance; *P<0.05, ** P<0.01, and *** P < 0.001.

We then tested the *ex vivo* anti-HIV-1 activity of IBP-CP24, N6 or IBP-CP24/N6 combination in the treated mice. As shown in Fig 9A and 9B, the sera from mice treated with the N6 (3.2 mg/kg)/IBP-CP24 (1.8 mg/kg) combination showed significantly higher inhibition (about 80- and 60-fold of its IC_50_ at 2 and 8 h post-injection, respectively) than N6 (3.2 mg/kg) alone (DF-IC_50_ = 44 and 38 at 2 and 8 h post-injection, respectively) and IBP-CP24 (1.8 mg/kg) alone (DF-IC_50_ = 35 and 16 at 2 and 8 h post-injection, respectively), while exhibited similar inhibitory activity as that of N6 at higher dose (5 mg/kg) with DF-IC_50_ of 90 and 64 at 2 and 8 h post-injection, respectively. The dose reduction effect of N6 possibly resulted from the synergistic effect of its combination with IBP-CP24. However, the sera from mice injected with N6 (3.2 mg/kg)/CP24 (1.8 mg/kg) exhibited significantly lower inhibitory activity than those from mice treated with N6 (3.2 mg/kg)/IBP-CP24 (1.8 mg/kg) combination, possibly because CP24 could not bind with N6 to improve its *in vivo* stability, thus being unable to act cooperatively with N6. These results suggest that *in vivo* binding of IBP-CP24 to N6 can increases the stability of the peptide and allows them to function in a synergistic manner. Therefore, the dose of bNAb N6 can be reduced when it is used in combination with IBP-CP24 *in vivo*.

## Discussion

The application of HIV-1 fusion inhibitors that target the viral membrane fusion step for HIV treatment was limited by their short half-life *in vivo* [9–10, 42–43]. Although methods, such as Fc/albumin fusion, have been tested extensively on proteins, most of these modifications interfere with the functions of the peptide inhibitors owing to the size of the fusion protein that results in steric hindrance [44–45]. To overcome this problem, we selected IBP as our target because it is smaller and simpler. Moreover, it can bind with the Fc of human IgG to stabilize the peptide.

In this study, we focused on IBP linked to the N-terminus of CP24 (IBP-CP24) because it showed more potent inhibitory activity than CP24 alone against laboratory-adapted HIV-1 strains IIIB (X4) and Bal (R5). Meanwhile, we also demonstrated that the inhibitory mechanism of CP24 was not affected by conjugated IBP, as IBP-CP24 could still bind with N36 and exhibit broader and better anti-HIV activity compared to CP24 alone. We demonstrated that IBP dramatically extended the plasma half-life of CP24 *in vivo*. Furthermore, pre-treatment of the humanized mice with IBP-CP24 could protect the mice against HIV-1 infection. Importantly, when co-injected with normal human IgG, the new fusion inhibitor IBP-CP24 could significantly decrease plasma viremia in mice with persistent HIV-1 infection. In addition, combining IBP-CP24 with a bNAb, such as N6, which targeting CD4-binding site in gp120, exhibited a synergistic effect. Overall, our results indicate that IBP-CP24 is a potent, long-lasting HIV-1 fusion inhibitor that can be used alone or in combination with a bNAb for prevention and treatment of HIV-1 infection.

Peptide drugs have been extensively studied for the treatment of virus infection and metabolic diseases [46–52], but consensus holds that the short half-life of peptides in plasma significantly limits their clinical use [7, 51, 53]. Several approaches have been tested to improve the serum half-life of peptide drugs, including chemical modifications (e.g., PEGylation) and conjugation with other proteins that are more stable in the serum. However, these methods have drawbacks, as demonstrated by studies of PEGylation. Many reports have illustrated that PEGylated liposomes, nanoparticles and proteins can elicit antibody responses against PEG, thus limiting their therapeutic efficacy or reducing the tolerance of PEGylated therapeutics [13–14, 54–55]. In addition, unexpected changes in pharmacokinetic behavior can occur with PEG-based carriers [56–58]. Meanwhile, PEG is not biodegradable, leading to toxicity concerns, such as the occurrence of vacuolization in kidney or liver cells [59–62]. IgG binding peptide (IBP) is an alternative method that can overcome these deficiencies by reversibly binding with human IgG Fc. Importantly, the simple structure and low molecular weight of IBP leads to potentially low, or no, antibody response against IBP, and it can be easily degraded.

Interestingly, the conjugation of IBP to the N-terminus of CP24 enhanced its inhibitory activity. This was a favorable, but unexpected outcome. In any case, we can attribute this phenomenon to IBP conjugation, which leads to the steric structural change of CP24, in turn enhancing the binding of the N-terminal MT hook of CP24 to the hydrophobic grooves of the gp41 NHR [20, 63]. This implies that further modifications of fusion peptide inhibitors might improve their function. This could also explain why IBP-CP24 exhibits increased binding affinity to IgG Fc compared to IBP itself, in which the fusion-induced structural change drives the exposure of more hydrophilic regions of IBP, resulting in better binding to the solvent C_H_2 and C_H_3 region of IgG Fc [19] and improving the stability of CP24 *in vivo*. Recently, some studies have shown that the average level of serum IgG in HIV-positive individuals is significantly higher than that in healthy controls [64–65]. This condition makes the application of IBP-CP24 even more favorable in HIV treatment, as more IgG can further stabilize IBP-CP24 in HIV patients, which could dramatically reduce the potential side effects caused by repetitive injection.

Combined anti-retroviral therapy (cART) suppresses viral replication and improves quality of life for those HIV-1-infected patients. However, cART is not curative and must be kept the whole lifetime. Moreover, drug resistant virus is developed in some cART-treated patients. Most recently, bNAb combinations have also been identified for their potential activity in reducing viremia and viral rebound in a humanized mouse model. Halper-Stromberg *et al*. have reported that combining the bNAbs 3BNC117, 10–1074, and PG16 that target CD4bs [66], V3 glycan [67], and V1V2 glycan [68], respectively, decreased viremia in about 50% of the mice and dramatically delayed virus rebound compared with mice administered with cART [69]. Therefore, bNAbs could significantly impact plasma viremia through recognition of HIV-1 Env [69]. However, high cost and complicated production of antibodies have slowed down further application of these antibodies for clinical use [70]. The synthesis of small-peptide inhibitors is relatively simple and fast compared to bNAbs; therefore, they can be used as a good alternative to reduce the cost of HIV treatment, especially in underdeveloped countries. Furthermore, since the binding of IBP-CP24 to human IgG Fc did not compromise the function of antibodies, combination of IBP-CP24 and a bNAb could further improve the therapeutic effects of bNAbs, allowing better control over chronic HIV infection and disease progression. It has recently been reported that the combination of attachment inhibitor BMS-626529 [71–72] and CD4bs bNAbs showed synergistic effect against virus [73]. We also demonstrated here that IBP-CP24 significantly enhanced the inhibitory activity of CD4bs bNAbs, *e*.*g*., N6 and VRC07, further confirming the potential of combinatorial therapy with both reagents.

In summary, conjugating IBP to the N-terminus of CP24, designated IBP-CP24, dramatically extended the serum half-life of CP24 *in vivo* by reversibly binding with IgG Fc and maintained broad and potent anti-HIV-1 activity of CP24 *in vitro*. Furthermore, IBP-CP24 protected humanized mice from HIV-1 infection, and significantly reduced plasma viremia in mice with chronic HIV-1 infection when co-administered with normal human IgG. Therefore, the advances made by IBP-CP24, as detailed in this work, show its potential for the development either as a prophylactic agent to prevent HIV-1 infection in high-risk populations or as a long-acting therapeutic medicine for the treatment of low-adherence AIDS patients. In addition, combining IBP-CP24 with a bNAb targeting CD4bs in gp120 (*e*.*g*., N6 and VRC07) or the MPER in gp41 (*e*.*g*., 10E8) exhibited synergistic effect. It is expected that in the combination, the bNAb may act as a biomissile carrying IBP-CP24. After the bNAb binds gp120, it makes the first strike to HIV-1. Then it releases IBP-CP24, which binds gp41 and makes the second strike to HIV-1. Therefore, combining IBP-CP24 with a bNAb may reduce the dose of the antibody and peptide (S5 Fig), thus the cost of the treatment. Therefore, IBP-CP24 has the potential to be further developed as a new HIV-1 fusion inhibitor-based, long-acting anti-HIV drug that can be used alone or in combination with a bNAb for treatment and prevention of HIV-1 infection.

## Materials and methods

### Ethics statement

Human peripheral blood mononuclear cells (PBMCs) were purchased from AllCell (Chicago, IL, USA). The protocol of monkey experiments was approved by the Institutional Animal Care and Use Committee of the Institute (IACUC) of Laboratory Animal Science, Chinese Academy of Medical Sciences (XJ17005). The experiments on rhesus monkeys were conducted following the Guide for the Care and Use of Laboratory Animals of the Institute of Laboratory Animal Science and the recommendations of the Weatherall report for the use of non-human primate (NHP), *i*.*e*., rhesus monkeys, in research. ICR and BALB/c mice were purchased from the Department of Laboratory Animal Science at Fudan University. The experiments on ICR and BALB/c mice were conducted under the ethical guidelines and approved by the Shanghai Public Health Clinical Center Animal Welfare and Ethics Committee at Fudan University (Approval No. 2017-A046–01). The New Zealand White rabbits were purchased from the Animal Center of Fudan University (Shanghai, China). The experiments on New Zealand White rabbits were conducted under the ethical guidelines and approved by Animal Center of Fudan University (License number DF-206). Human fetal livers were obtained from medically indicated or elective termination of pregnancies through a non-profit intermediary working with outpatient clinics (Advanced Bioscience Resources, Alameda, CA). Written informed consent from the maternal donor was obtained in all cases under regulations governing the clinic. The protocol for the use of humanized mice studies were approved by the University of North Carolina Institutional Animal Care and Use Committee (IACUC ID: 17–051.0). All humanized mice studies were conducted following NIH guidelines for housing and care of laboratory animals.

### Peptides, cells, viruses, and antibodies

Peptides (Fig 1A) were synthesized by a standard solid-phase at KareBay Biochem, Inc., as described previously [74–75]. The purities of peptides were more than 90%, as determined by reversed-phase high-pressure liquid chromatography (HPLC). The peptides were dissolved in dimethyl sulfoxide (DMSO) or PBS. The concentrations of peptides were measured by UV absorbance, and molar-extinction coefficient of peptides was calculated based on tryptophan and tyrosine residues [63]. 293T cells were obtained from the American Type Culture Collection (ATCC; Manassas, VA, USA). Human peripheral blood mononuclear cells (PBMCs) were purchased from AllCell (Chicago, IL, USA). MT-2 cells, CEMx174 5.25 M7 cells, HIV-1 IIIB chronically infected H9 (H9/HIV-1_IIIB_) cells, TZM-bl cells, U87 CD4^+^ CCR5^+^ cells, HIV-1 strains, and HIV neutralizing antibodies VRC07, 10E8 and N6 were obtained from the NIH AIDS Reagent Program.

### Circular dichroism (CD) spectroscopy

CD spectroscopy was conducted as previously described [76]. Briefly, a CHR peptide was incubated with NHR peptide N36 at concentration of 10 μM at 37 °C for 30 min. The separated C-peptides were also measured. The CD spectra were measured on a Jasco spectropolarimeter (model J-815; Jasco, Inc., Easton, MD, USA), using a 1-nm bandwidth with a 1-nm step resolution from 195 to 260 nm at room temperature. The spectra were corrected by subtraction of PBS. The α-helical content was calculated from the CD signal by dividing the mean residue ellipticity [θ] at 222 nm by the value expected for 100% helix formation (-33,000 deg·cm^2^·dmol^-1^) [77]. The thermal denaturation experiment was performed by monitoring the changes in ellipticity [θ] at 222 nm at increasing temperature from 20 to 98 °C at a rate of 1.2 °C/min; data were acquired at a 1-nm bandwidth at 222 nm at a frequency of 0.25 Hz. The melting curve was smoothed, and the midpoint of the thermal unfolding transition (T_m_) value was taken as the maximum of the derivative d[θ] _222_/dt. T_m_ value was detected at a peptide concentration of 10 μM in PBS buffer.

### Inhibition of HIV-1 infection by peptides

The inhibitory activities of peptides against HIV-1 IIIB virus (subtype B, X4), HIV-1 Bal (subtype B, R5), HIV-1 clinical strains, and T20-resistant strains were evaluated, as previously described [76, 78–79]. Briefly, 1×10^5^/mL MT-2 or M7 cells were infected with different HIV-1 viruses at 100 TCID50 (50% tissue culture infective dose) in the presence or absence of peptides at graded concentrations overnight. Then the culture supernatants were replaced with equal volume of fresh medium. On the fourth day post-infection, supernatants were collected and mixed with equal volume of 5% Triton X-100. The p24 antigen was determined by an enzyme-linked immunosorbent assay (ELISA), as previously described [80]. The IC_50_ values were calculated with CalcuSyn software [35–36], and inhibition curves of best fit were generated with GraphPad Prism.

### HIV-1-mediated cell-cell fusion

The inhibitory activity of peptides against cell-cell fusion between H9/HIV-1_IIIB_ and MT-2 cells was detected, as described previously [3, 81]. Briefly, H9/HIV-1_IIIB_ cells were labeled with a fluorescent reagent, Calcein AM (Molecular Probes, Inc., Eugene, Oregon), and then incubated with MT-2 cells (ratio = 1:10) in a 96-well plate at 37 °C for 2 h in the presence of a test peptide at graded concentrations or PBS as a control. The fused and unfused Calcein-labeled HIV-1-infected cells were counted under an inverted fluorescence microscope (Zeiss, Germany). Three fields of each well were counted and the percentage of inhibition of cell fusion by peptides was calculated as described previously [81]. The IC_50_ values of fusion were calculated using the CalcusSn software [36] and the inhibition curves of best fit were generated with GraphPad Prism.

### Inhibition of 6-HB formation by peptides

The inhibitory activity of peptides on 6-HB formation was measured by the sandwich ELISA method, as previously described [82]. Briefly, a 96-well polystyrene plate (Costar, Corning Inc., Corning, NY) was coated with rabbit anti-gp41 (NY-364) (2 μg/mL in 0.1 M Tris, pH 8.8). A test peptide (CP24, IBP or IBP-CP24) at graded concentrations was incubated with N36 (1 μM) at room temperature for 30 min and then incubated with C34 (1 μM) at the same condition. The mixture was added to the NY-364-coated plate, followed by incubation at room temperature for 60 min and washed three times with a washing buffer (PBS containing 0.1% Tween 20). The monoclonal antibody NC-1(1 μg/mL) was added to the plate and incubated at room temperature for 60 min, followed by washing three times. Rabbit anti-mouse IgG-HRP (Sigma) (1:3000 diluted) was added to each well and incubated at 37 °C for 1 h. The substrate 3, 3’, 5, 5’-tetramethylbenzidine (TMB; Sigma) was then added sequentially. Absorbance at 450 nm was measured using an ELISA reader (Ultra 384; Tecan, Research Triangle Park, NC). The percent inhibition by the peptides and the IC_50_ were calculated, as previously described [78].

### ELISA to evaluate IgG binding to peptides

The ability of human Immunoglobulin G (IgG) (Thermo Fisher Scientific) or rhesus monkey IgG (purified from sera of rhesus monkeys) to bind to IBP, CP24 or IBP-CP24 was evaluated by ELISA. Each well of a 96-well plate was coated with 50 μL of a 5 μg/mL solution of IBP, CP24 and IBP-CP24 in 0.1 M coating buffer at 4 °C overnight. The plate was washed three times with 150 μL phosphate-buffered saline (PBS) containing 0.1% Tween 20 (PBS-T) and blocked for two hours at room temperature with 150 μL of a 2% fat free milk solution in PBS. Next, different concentrations of human or rhesus monkey IgG were added to the peptide-coated plate and incubated at 37 °C for 1 h. Rabbit anti-human IgG-HRP (1:3000 diluted) was added to each well and incubated at 37 °C for another 1 h. Afterwards, the bound IgG-HRP was detected by addition of the HRP substrate TMB. The reaction was terminated by addition of 1 M H_2_SO_4_ 50 μL. Absorbance at 450 nm was measured using an ELISA reader (Ultra 384; Tecan, Research Triangle Park, NC).

### Detection of CP24- or IBP-CP24 specific antibody in plasmas of rhesus monkeys using an indirect and a sandwich ELISA

We first used an indirect ELISA to determine whether the *in vivo* application of IBP-CP24 can induce IBP-CP24-specific antibody, three groups of rhesus monkeys were intravenously administered with PBS, CP24 (10 mg/kg), or IBP-CP24 (10 mg/kg), respectively, daily for one month. The plasma samples were collected at the indicated time points for detecting CP24 or IBP-CP24-specific antibody using an indirect ELISA. Briefly, we coated the wells of a 96-well plate with 50 μL of CP24 or IBP-CP24 (5 μg/mL) in 0.1 M coating buffer at 4 °C overnight, washed the plate three times with 150 μL PBS-T, blocked the plate with 150 μL of a 2% fat free milk solution in PBS for 2 h at room temperature, and added the serially 10-fold diluted plasma of the PBS- or peptide-treated rhesus monkeys. The CP24 or IBP-CP24-specific IgG was detected as described above.

We then adapted a captured peptide ELISA as previously described [83], to detect CP24- or IBP-CP24 specific antibody in the rhesus monkeys' plasma. In brief, we coated the wells of a 96-well plate with 50 μL of streptavidin (sigma) (1 μg/mL) in 0.1 M coating buffer at 4 °C overnight, washed the plate three times with 150 μL PBS-T, blocked the plate with 150 μL of 1% BSA in PBS for 2 h at room temperature, and added biotinylated CP24 or IBP-CP24 peptides. After incubation at 4 °C for 2 h and washes with PBS-T for three times, we added the serially 10-fold diluted plasma of the PBS- or peptide-treated rhesus monkeys. Goat Anti-Monkey IgG H&L (HRP) (Abcam) were incubated for 1 h at 37 °C and washed for five times. The CP24 or IBP-CP24-specific IgG was detected as described above.

### Isothermal titration calorimetry (ITC)

ITC assay was performed with an ITC microcalorimeter instrument (NANO ITC, TA Instruments, Lindon, UT) as described previously [84]. In brief, 300 μM peptides were dissolved in PBS and injected into the chamber containing 30 μM of rhesus monkey IgG. The experiments were carried out at 25 °C. The time between injections was 300 s, and the stirring speed was 300 rpm. The heats of dilution were determined in control experiments by injecting peptides into PBS and then subtracting from the heats produced in the corresponding peptide-IgG binding experiments. Data acquisition and analysis were performed using the Origin software, and the best fitting curves were generated with GraphPad Prism.

### Pharmacokinetic studies in rhesus monkeys

Five rhesus monkeys were administered intravenously with a single dose of 10 mg/kg CP24 (n = 2) or IBP-CP24 (n = 3). Serial blood samples were collected from monkeys that received CP24 or IBP-CP24 before injection and at 2, 4, 6, 24, 72 and 144 h post-injection in tubes containing EDTA. The samples were then centrifuged (3200 rpm for 10 min at 4 °C), and plasma was aliquoted and kept frozen until analysis.

### Double antibody sandwich ELISA method for pharmacokinetic analysis of plasma samples

A 96-well polyclonal microplate was coated with rabbit anti-IBP-CP24 polyclonal antibody (purified from IBP-CP24 immune rabbit serum) in carbonate buffer (pH 9.6) and incubated at 4 °C overnight. After being coated, the plate was washed three times with PBS-T to remove the excess antibody. The remaining active sites on the plate were blocked by the addition of 150 μL 2% milk for 2 h at 37 °C. The serially diluted IBP-CP24 or CP24 standard solution, negative control, and diluted unknown samples were added to appropriate wells and incubated at 37 °C for 1 h; each sample, including controls, was tested in triplicate. After the plate was washed as described above, mouse anti-IBP-CP24 or CP24 serum (collected from BALB/c mice immunization with IBP-CP24 or CP24) at a dilution of 1:1000 was added and incubated at 37 °C for another 1 h. Then, HRP-labeled rabbit anti-mouse IgG (Sigma, USA) incubated at 37 °C for 1 h. The substrate TMB reagent were added and incubated for 5 min, resulting in the development of a blue color. Color development was stopped by the stop solution (1M H_2_SO_4_ 50 μL/well). Absorbance at 450 nm was determined by an ELISA reader (Ultra 384, Tecan; Tecan, Research Triangle Park, NC). Concentrations of IBP-CP24 and CP24 in the unknown samples were calculated by interpolation from the IBP-CP24 and CP24 standard curves, respectively. The half-life of IBP-CP24 and CP24 was calculated by using PK Solver Excel to obtain pharmacokinetic parameters.

### Detection of the ex vivo anti-HIV-1 activity and plasma half-life of the peptides or bNAbs tested

The *ex vivo* anti-HIV-1 activity of a peptide or a bNAb or their combination in serum samples from mice treated with the peptide or bNAb was detected as described previously [23–26]. Briefly, BALB/c mice were peritoneally administered with N6 at 5 mg/kg, N6 at 3.2 mg/kg, IBP-CP24 at 1.8 mg/kg, N6 (3.2 mg/kg)/IBP-CP24 (1.8 mg/kg) combination, N6 (3.2 mg/kg)/CP24 (1.8 mg/kg) combination, or PBS (as a control), respectively. Sera were collected from these mice at 2 and 8 h post-injection and tested for their inhibitory activity against HIV-1_IIIB_ infection as described above. The dilution fold of the serum causing 50% inhibition (DF-IC_50_) was calculated. The *ex vivo* anti-HIV-1 activity of the peptide IBP-CP24 in the plasma samples from rhesus monkeys treated with the peptide was also evaluated using this method. Based on the DF-IC_50_ values calculated, the concentration of IBP-CP24 in the plasma samples was estimated and its plasma half-life and other pharmacokinetic parameters were calculated using MODFIT software as previously described [26, 85].

### Inhibition of pseudotyped HIV-1 infection

HIV-1 pseudoviruses were generated, as described previously [86]. Briefly, 293T cells were cotransfected with plasmids encoding HIV-1 envelope and luciferase-expressing HIV-1 genome using VigoFect reagent (Vigorous Biotech, Beijing, China). The supernatants were replaced with fresh DMEM with 10% FBS at 12 h post-transfection and collected at 48 h post-transfection and stored at -80 °C. The antiviral activities of peptides were determined using U87 CD4^+^ CCR5^+^ cells. Different concentrations of peptide mixed with 100 TCID50 virus and U87 CD4^+^ CCR5^+^ cells (10^4^/well). Changed fresh medium and cultured for additional 48 h at 37 °C, and the luciferase activity was measured by using a luciferase kit (Promega, Madison, WI) and an Ultra 384 luminometer (Tecan).

### Construction of humanized mice Nod-rag-gC

NRG (NOD Rag2^-/-^ γc^-/-^) mice were obtained from the Jackson Laboratory. All mice were housed and bred in a specific pathogen–free conditions at the University of North Carolina. Humanized NRG mice with a functional human immune system were generated, as previously reported [87]. Human CD34^+^ cells were isolated from fetal liver tissues obtained from the Advanced Bioscience Resources, Alameda, CA. CD34^+^ cells were injected into the liver of NRG mice. Human immune cell engraftment was detected by flow cytometry around 12 weeks after transplantation of human CD34^+^cells (NRG-hu HSC mice).

### Prophylactic efficacy of IBP-CP24 against HIV-1 challenge in humanized mice

For prophylactic study, humanized NRG mice were administered intraperitoneally with PBS, 20 mg/kg CP24 or 20 mg/kg IBP-CP24 before virus challenge. The mice were challenged with HIV-1 JR-CSF (5 ng p24/mouse) through retro-orbital injection at 2 h post-treatment [88]. Then the mice were treated with PBS or peptides at 6 hours post-infection and once every day for 3 consecutive days. Plasma samples were obtained for viral load determination at days 0, 7, 14, 21 and 28 post-infection.

### Therapeutic efficacy of IBP-CP24 in humanized mice with established HIV-1 infection

Humanized mice were inoculated with HIV-1 JR-CSF (10 ng p24/mouse) through retro-orbital injection and randomly assigned to 2 groups (4 mice in each group). Chronically infected humanized mice were intraperitoneally injected with 200 μL of CP24 (20 mg/kg) or IBP-CP24 (20 mg/kg) once daily for 14 days from day 35 after virus inoculation, and then mice were given inhibitors twice daily for 21 days. Since human IgG can persist with stability (t_1/2_ = 10 days) *in vivo* [89], mice were intraperitoneally injected with 40 mg/kg human IgG twice every week to stabilize inhibitors *in vivo* during treatment.

### Measurement of plasma HIV-1 RNA by qRT-PCR

HIV-1 genomic RNA was purified from plasma by using the QIAamp Viral RNA Mini Kit. The RNA was reverse-transcribed and quantitatively detected by real-time PCR with the TaqMan Fast Virus 1-Step PCR kit (Thermo Fisher Scientific). We used the forward primer 5’-GGTGCGAGAGCGTCAGTATTAAG-3’ and the reverse primer 5’-AGCTCCCTGCTTGCCCATA-3’ to detect the HIV gag gene. The probe (FAM-AAAATTCGGTTAAGGCCAGGGGGAAAGAA-QSY7) used for detection was ordered from Applied Biosystems, and the reactions were set up following the manufacturer’s guidelines and were run on the QuantStudio 6 Flex PCR system (Applied Biosystems). The limit of detection of each real-time PCR was 4 copies per reaction. Accordingly, owing to the relatively small volume of plasma in mice (about 50–100 μL total blood), the limit of detection was 400 copies/mL plasma. The copy numbers below the detectable limit were set as 1. Triplicate reactions were performed for each sample.

### Cell-associated HIV-1 DNA detection

To evaluate cell-associated HIV-1 DNA, total DNA was extracted from mouse spleen and bone marrow cells using the DNeasy Mini Kit (Qiagen). HIV-1 DNA was quantified by real-time PCR. DNA from serial dilutions of ACH2 cells, which contain 1 copy of the HIV genome in each cell, was used to create a standard curve.

### Cell-associated HIV-1 RNA detection

To detect cell-associated HIV-1 RNA, total cell RNA was extracted from mouse spleen and bone marrow cells using the RNeasy Plus Mini Kit (Qiagen). The RNA was reverse-transcribed to cDNA and then quantified by real-time PCR, as described above. DNA from serial dilutions of ACH2 cells was used to generate a standard curve.

### ADCC assay

H9/HIV-1_IIIB_ cells expressing HIV-1 Env (2.5 × 10^4^ cells) as the target cells were seeded into a 96-well plate (25 μL/well), and the mixture of a serially diluted inhibitors and 10E8 (1.5 μg/mL) was added to the culture plate. PBMC (AllCells) as effector cells (2.5 × 10^4^ cells in 25 μL) were cocultured with the mixture-treated target cells at 37 °C overnight, and the luciferase activity was measured using the CytoTox-ONE Homogeneous Membrane Integrity Assay (Promega).

### Cytotoxicity assay

The potential cytotoxicity of peptides on MT-2 and M7 cells was measured by using the Cell Counting Kit-8 according to the manufacturer’s instructions (CCK-8, Dojindo Laboratories, Kumamoto, Japan). Briefly, 100 μL of a peptide at graded concentrations were added to equal volumes of cells (10^4^ cells/well) in a 96-well plate. Cell viability was evaluated using the CCK-8 kit after incubation at 37 °C for 2 days. Incubation with CCK-8 reagents at 37 °C for another 4 h, the absorbance at 450 nm was measured with an ELISA reader, and the percentage of cytotoxicity was calculated.

### Safety of IBP-CP24 for ICR mice

Fifteen ICR mice (6–8 weeks old) were assigned randomly to 4 groups and injected intraperitoneally with 10 mg/kg CP24 (*n* = 4), 10 mg/kg IBP-CP24 (*n* = 4), 100 mg/kg IBP-CP24 (*n* = 4), or PBS (*n* = 3) as control. Body weight changes of mice at different time points were monitored. Creatinine and ALT in the sera were measured by using the creatinine and ALT assay kits (NJJCBIO, Nanjing, China) before injection and 5 h, 1, and 4 days post-injection, respectively. All mice were sacrificed at day 15 post-injection, and the livers and kidneys were collected for haematoxylin and eosin (H&E) staining.

### Detection of inhibitory activity of bNAb and IBP-CP24 tested alone or in combination

To evaluate the potential synergistic effect, bNAb and IBP-CP24 were mixed at the indicated molar concentration ratio, whereas bNAb alone and IBP-CP24 alone were included as controls. The samples were serially diluted and tested for their inhibitory activity on HIV-1 infection as described above. The data were analyzed for synergistic effect by calculating the combination index (CI) with the CalcuSyn software [36]. CI values of <0.95 and >1.05 indicate synergy and antagonism, respectively. CI <0.1, 0.1–0.3, 0.3–0.7, 0.7–0.85, and 0.85–0.90 indicate very strong synergism, strong synergism, synergism, moderate synergism, and slight synergism, respectively [35]. Fold of dose reduction was calculated with the ratio of concentrations of inhibitor testing alone and in combination.

## Supporting information

S1 Table Estimated Kd of IBP-CP24 binding to human IgG and rhesus monkey IgG. (DOCX)

S2 Table Pharmacokinetic parameters of CP24 and IBP-CP24 in rhesus monkeys. (DOCX)

S1 Fig Ex vivo anti-HIV-1 activity and concentration of peptides in plasma samples from peptides treated monkeys. (DOCX)

S2 Fig Inhibitory activities of CP24 and IBP-CP24 against HIV-1 JR-CSF infection. (DOCX)

S3 Fig *In vivo* evaluation of the therapeutic efficacy of CP24 and IBP-CP24. (DOCX)

S4 Fig Detection of CP24 or IBP-CP24-specific antibody response in the CP24- or IBP-CP24-treated rhesus monkeys by a captured peptide ELISA. (DOCX)

S5 Fig Combinatorial use of bNAb N6 and IBP-CP24 for inhibition of HIV-1 infection. (DOCX)
