# Supplementary material for: IgG Fc-binding motif-conjugated HIV-1 fusion inhibitor exhibits improved potency and in vivo half-life: Potential application in combination with broad neutralizing antibodies
Source: PLoS Pathog. 2019 Dec 5;15(12):e1008082. doi: 10.1371/journal.ppat.1008082 (PMC6894747; doi:10.1371/journal.ppat.1008082)
Supplement: S1 Table — (DOCX) [file ppat.1008082.s001.docx]

**S1 Table.** Estimated dissociation constants (Kd) of IBP-CP24 binding to human IgG or rhesus monkey IgG.

| **Complex** | **Kd (M)** |
| --- | --- |
| **IBP-CP24/human IgG** | 1.77E-07 ± 4.24E-08 |
| **IBP-CP24/rhesus monkey IgG** | 5.57E-07 ± 1.26E-07 |

The data was fitted to the “one site – specific binding” using GraphPad Prism. The standard error (SE) is given for a sample size of three replicates per data point.
