# Supplementary material for: IgG Fc-binding motif-conjugated HIV-1 fusion inhibitor exhibits improved potency and in vivo half-life: Potential application in combination with broad neutralizing antibodies
Source: PLoS Pathog. 2019 Dec 5;15(12):e1008082. doi: 10.1371/journal.ppat.1008082 (PMC6894747; doi:10.1371/journal.ppat.1008082)
Supplement: S2 Table — (DOCX) [file ppat.1008082.s002.docx]

**S2 Table.** Pharmacokinetic parameters of CP24 and IBP-CP24 in rhesus monkeys.

| **Parameter** | **CP24** | **IBP-CP24** |
| --- | --- | --- |
| t_1/2_ (h) | 1.37 | 44.81 |
| Tmax (h) | 2 | 4 |
| Cmax (μg/mL) | 77.82 | 76.23 |
| AUC 0-t (μg/mL*h) | 237.88 | 3610.65 |
| MRT 0-inf_obs (h) | 3.17 | 61.39 |
