# Supplementary material for: IgG Fc-binding motif-conjugated HIV-1 fusion inhibitor exhibits improved potency and in vivo half-life: Potential application in combination with broad neutralizing antibodies
Source: PLoS Pathog. 2019 Dec 5;15(12):e1008082. doi: 10.1371/journal.ppat.1008082 (PMC6894747; doi:10.1371/journal.ppat.1008082)

**S1 Fig. Ex vivo anti-HIV-1 activity and concentration of peptides in plasma samples from peptides treated monkeys.** (A) Anti-HIV-1 IIIB activity of the plasma samples collected from monkeys at different time points. (B) Estimated the concentration of the active peptides in the plasma samples.


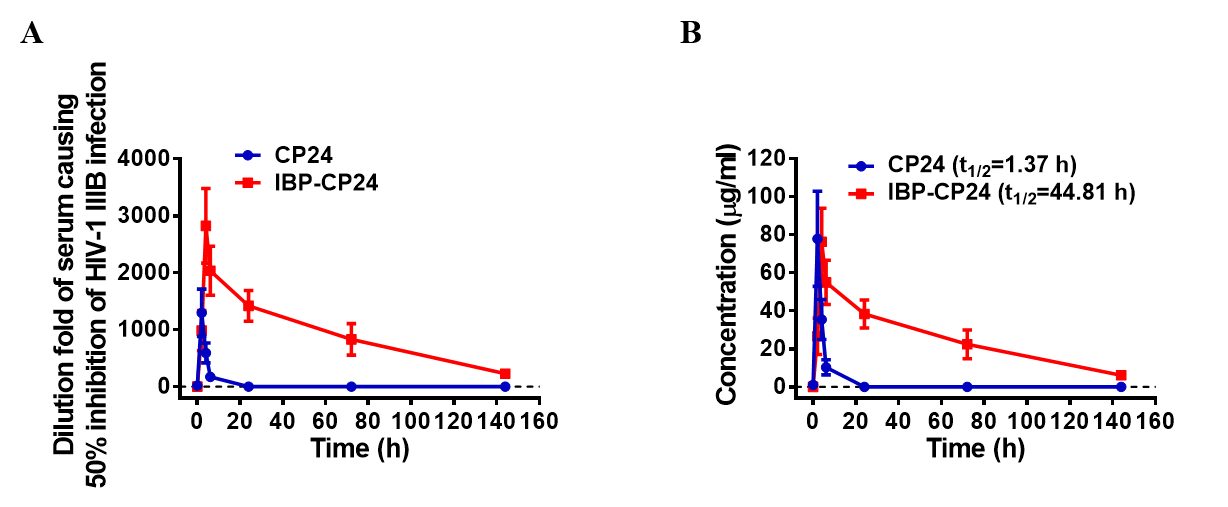

Supplement: S1 Fig — (DOCX) [file ppat.1008082.s003.docx]
