# Supplementary material for: IgG Fc-binding motif-conjugated HIV-1 fusion inhibitor exhibits improved potency and in vivo half-life: Potential application in combination with broad neutralizing antibodies
Source: PLoS Pathog. 2019 Dec 5;15(12):e1008082. doi: 10.1371/journal.ppat.1008082 (PMC6894747; doi:10.1371/journal.ppat.1008082)

**S2 Fig. Inhibitory activities of CP24 and IBP-CP24 against HIV-1 JR-CSF infection.** Each sample was detected in triplicate, and the data are presented as mean ± SD.


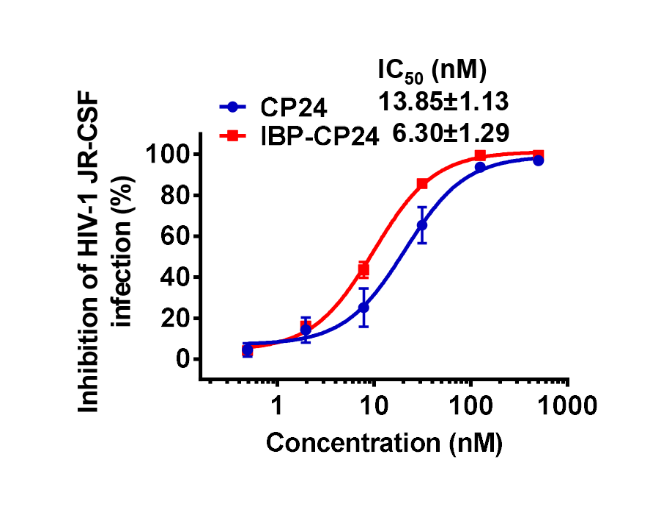

Supplement: S2 Fig — (DOCX) [file ppat.1008082.s004.docx]
