# Supplementary material for: IgG Fc-binding motif-conjugated HIV-1 fusion inhibitor exhibits improved potency and in vivo half-life: Potential application in combination with broad neutralizing antibodies
Source: PLoS Pathog. 2019 Dec 5;15(12):e1008082. doi: 10.1371/journal.ppat.1008082 (PMC6894747; doi:10.1371/journal.ppat.1008082)

**S3 Fig. *In vivo* evaluation of the therapeutic efficacy of CP24 and IBP-CP24 coinjected with human IgG in HIV-1-infected humanized mice.** (A) Schematic diagram of the therapeutic experimental design. Humanized mice were infected with HIV-1 at day -35. At day 0, the mice received CP24 or IBP-CP24 treatment daily through day14 (purple arrows). Beginning from day 14 through day 35, the mice received CP24 or IBP-CP24 treatment twice a day (red arrows). All the mice were injected with human IgG twice every week. (B and C) The level of plasma HIV-1 RNA was monitored at the indicated time points in mice treated with CP24 (B) or IBP-CP24 (C).


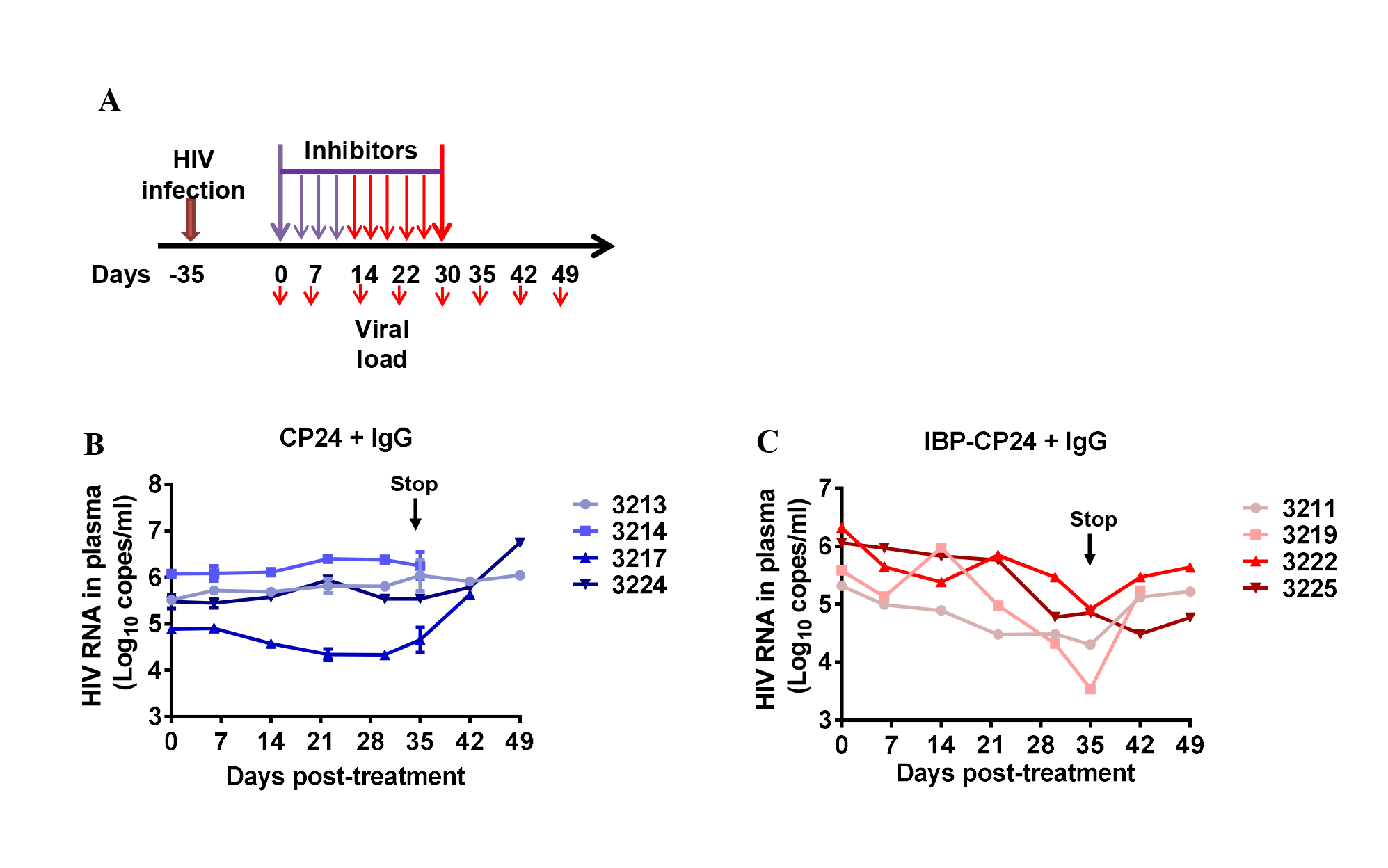

Supplement: S3 Fig — (DOCX) [file ppat.1008082.s005.docx]
