# Supplementary material for: IgG Fc-binding motif-conjugated HIV-1 fusion inhibitor exhibits improved potency and in vivo half-life: Potential application in combination with broad neutralizing antibodies
Source: PLoS Pathog. 2019 Dec 5;15(12):e1008082. doi: 10.1371/journal.ppat.1008082 (PMC6894747; doi:10.1371/journal.ppat.1008082)

**S4 Fig.** **Detection of CP24 or IBP-CP24-specific antibody response in the CP24- or IBP-CP24-treated rhesus monkeys by a captured peptide ELISA.** The level of CP24 or IBP-CP24-specific IgG in the plasma of rhesus monkeys intravenously administered with PBS, CP24 or IBP-CP24 was detected by a captured peptide ELISA, in which streptavidin was immobilized for capture of the biotinylated CP24 or IBP-CP24 peptides. The data were presented as mean ± SD.


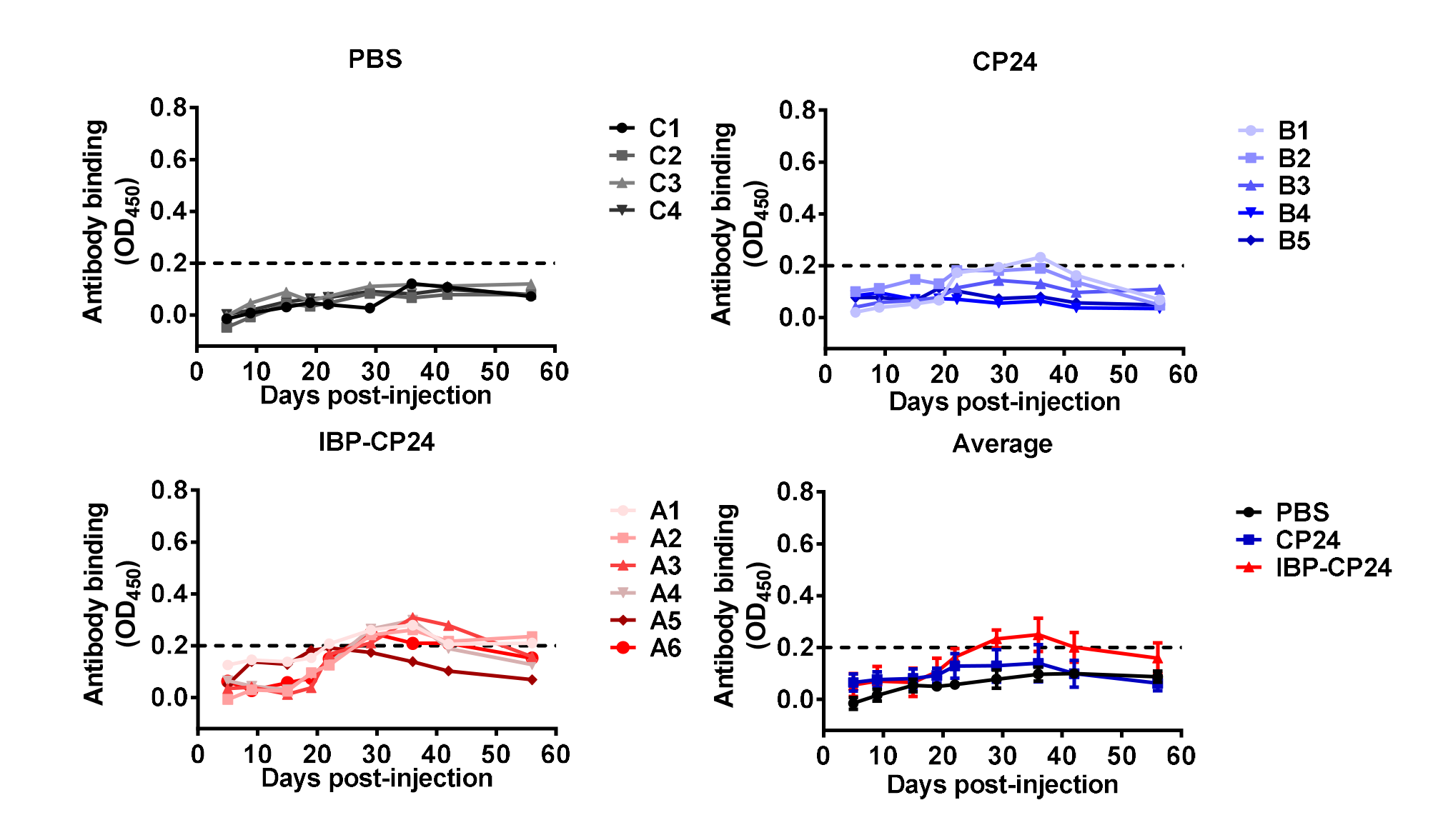

Supplement: S4 Fig — (DOCX) [file ppat.1008082.s006.docx]
