# Supplementary material for: IgG Fc-binding motif-conjugated HIV-1 fusion inhibitor exhibits improved potency and in vivo half-life: Potential application in combination with broad neutralizing antibodies
Source: PLoS Pathog. 2019 Dec 5;15(12):e1008082. doi: 10.1371/journal.ppat.1008082 (PMC6894747; doi:10.1371/journal.ppat.1008082)

**S5 Fig. Combinatorial use of bNAb N6 and IBP-CP24 peptide for inhibition of HIV-1 infection.** The gp120-targeting bNAb N6 may act as a biomissile carrying gp41-targeting IBP-CP24 peptide. After N6 binds to gp120, it makes the first strike to HIV-1. Then it releases IBP-CP24 peptide, which makes the second strike to HIV-1. Therefore, combinational use of IBP-CP24 and bNAb is expected to have synergistic and complementary effect against HIV-1.


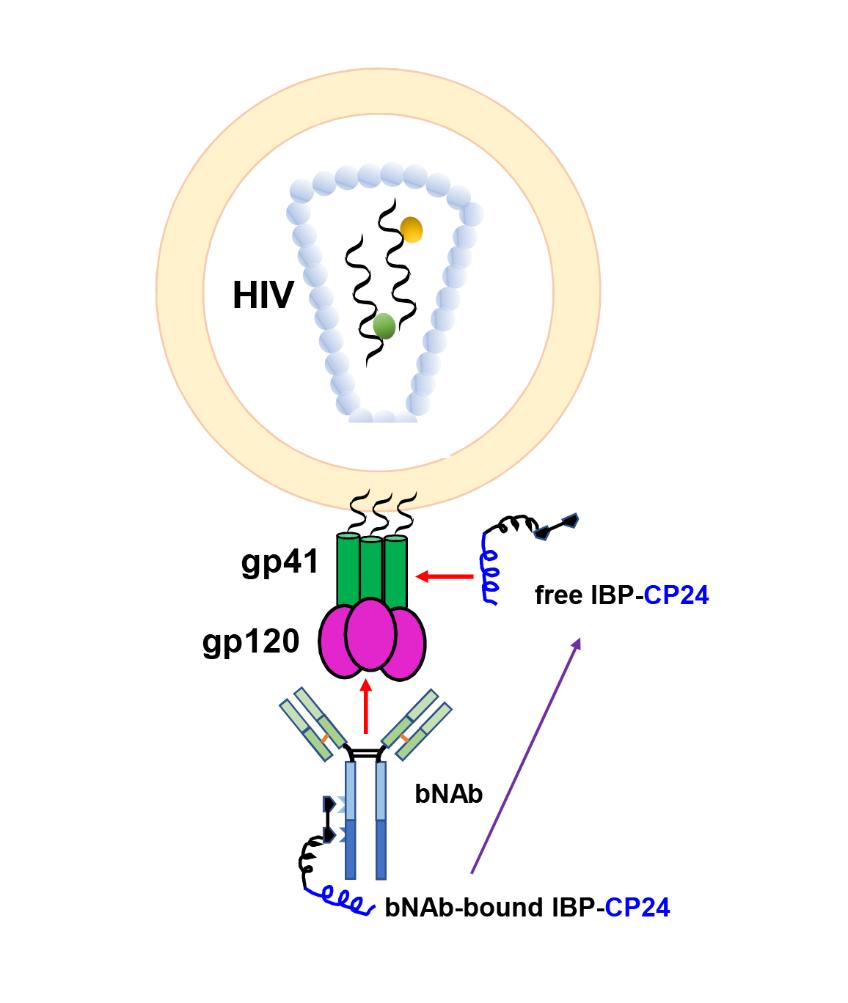

Supplement: S5 Fig — (DOCX) [file ppat.1008082.s007.docx]
